# Cerebrospinal fluid and venous biomarkers of shunt-responsive idiopathic normal pressure hydrocephalus: a systematic review and meta-analysis

**DOI:** 10.1007/s00701-022-05154-5

**Published:** 2022-03-01

**Authors:** Santhosh G. Thavarajasingam, Mahmoud El-Khatib, Kalyan V. Vemulapalli, Hector A. Sinzinkayo Iradukunda, Joshua Laleye, Salvatore Russo, Christian Eichhorn, Per K. Eide

**Affiliations:** 1 Faculty of Medicine, Imperial College London, London, UK; 2 Department of Neurosurgery, Imperial College Healthcare NHS Trust, London, UK; 3 Division of Internal Medicine, University Hospital Basel, Basel, Switzerland; 4 Department of Neurosurgery, Oslo University Hospital, Rikshospitalet, Oslo Norway; 5 Faculty of Medicine, Institute of Clinical Medicine, University of Oslo, Oslo, Norway

**Keywords:** Diagnosis, Normal pressure hydrocephalus, Predict, Shunt response, iNPH, Biomarker, Tau

## Abstract

**Background:**

Idiopathic normal pressure hydrocephalus (iNPH) is a neurodegenerative disease and dementia subtype involving disturbed cerebrospinal fluid (CSF) homeostasis. Patients with iNPH may improve clinically following CSF diversion through shunt surgery, but it remains a challenge to predict which patients respond to shunting. It has been proposed that CSF and blood biomarkers may be used to predict shunt response in iNPH.

**Objective:**

To conduct a systematic review and meta-analysis to identify which CSF and venous biomarkers predict shunt-responsive iNPH most accurately.

**Methods:**

Original studies that investigate the use of CSF and venous biomarkers to predict shunt response were searched using the following databases: Embase, MEDLINE, Scopus, PubMed, Google Scholar, and JSTOR. Included studies were assessed using the ROBINS-I tool, and eligible studies were evaluated utilising univariate meta-analyses.

**Results:**

The study included 13 studies; seven addressed lumbar CSF levels of amyloid-β 1–42, nine studies CSF levels of Total-Tau, six studies CSF levels of Phosphorylated-Tau, and seven studies miscellaneous biomarkers, proteomics, and genotyping. A meta-analysis of six eligible studies conducted for amyloid-β 1–42, Total-Tau, and Phosphorylated-Tau demonstrated significantly increased lumbar CSF Phosphorylated-Tau (− 0.55 SMD, *p* = 0.04) and Total-Tau (− 0.50 SMD, *p* = 0.02) in shunt-non-responsive iNPH, though no differences were seen between shunt responders and non-responders for amyloid-β 1–42 (− 0.26 SMD, *p* = 0.55) or the other included biomarkers.

**Conclusion:**

This meta-analysis found that lumbar CSF levels of Phosphorylated-Tau and Total-Tau are significantly increased in shunt non-responsive iNPH compared to shunt-responsive iNPH. The other biomarkers, including amyloid-β 1–42, did not significantly differentiate shunt-responsive from shunt-non-responsive iNPH. More studies on the Tau proteins examining sensitivity and specificity at different cut-off levels are needed for a robust analysis of the diagnostic efficiency of the Tau proteins.

**Supplementary Information:**

The online version contains supplementary material available at 10.1007/s00701-022-05154-5.

## Introduction

Idiopathic normal pressure hydrocephalus (iNPH) is a neurodegenerative disease and subtype of dementia incorporating disturbed cerebrospinal fluid (CSF) homeostasis, first described in 1965 [2]. The clinical symptoms include gait ataxia, cognitive decline (dementia), urinary incontinence, and apathy [67] that may improve following CSF diversion (shunt) surgery. It remains an obstacle, however, that even though affected individuals fulfil the diagnostic criteria of probable iNPH according to the American-European [62] or Japanese [35, 56] guidelines, likely response to shunt surgery cannot be predicted from fulfilling the current diagnostic criteria. Therefore, the guidelines also differentiate between shunt-responsive and shunt-non-responsive iNPH. As a supplement to the guidelines, various predictors of shunt-responsive iNPH have been introduced [49]. The most common supplemental tests include imaging biomarkers of ventriculomegaly and CSF disturbance [3, 23, 75] biomarkers of CSF pressure dynamics (infusion tests and intracranial pressure (ICP) measures) [16, 24, 77] and clinical assessment following small (tap test) [77] or large (extended lumbar drain) [50] volume CSF diversion. Since the sole treatment option, shunt surgery, requires surgical intervention in the brain of affected individuals with a definite risk of severe complications [27, 47, 55, 62], there is a great need for identifying biomarkers of shunt-responsive iNPH [56, 71]. In addition, the occurrence of iNPH may be higher than previously assumed and may even affect several million people in Europe alone [5, 10, 36]. These figures also call for less invasive predictors of shunt-responsive iNPH.

There is a close overlap between iNPH and Alzheimer’s disease (AD) as both conditions present with abnormal deposition in the brain of toxic by-products of cerebral metabolism, such as amyloid-beta 1–42 (amyloid-β 1–42) and Tau [42]. Evidence from brain tissue examination even suggests that iNPH may be a model disease of Alzheimer’s disease [44]. Others have shown that comorbid Alzheimer’s disease is a strong predictor of shunt non-responsive iNPH [7, 8, 28, 29]. More recently, it was suggested that a final common pathway to dementia disease may be the pathological cerebral aggregation of toxic by-products of brain metabolism caused by impaired cerebral clearance of these waste products, e.g., deposition of amyloid-β 1–42 and Tau in Alzheimer’s disease and α-synuclein in Parkinson’s disease [57]. Due to the close association between iNPH and other dementia diseases such as Alzheimer’s disease and Parkinson’s disease, levels of biomarkers of neurodegeneration in CSF or blood could be used to differentiate shunt-responsive from shunt-non-responsive iNPH. Accordingly, the biomarkers Total-Tau (T-Tau) and amyloid-β 1–42 were previously hypothesised to aid in differentiating between Alzheimer’s disease and iNPH [37, 38]. Other biomarkers such as Phosphorylated-Tau (P-Tau) [12], interleukins [45], and neurofilament triplet protein (NFL) [4, 73] were suggested to participate in the evolvement of hydrocephalus and other neurological conditions. In line with this, the most recent guidelines for the management of iNPH patients recommend CSF assessment for all suspected iNPH patients [56].

To this end, there have been three systematic reviews investigating the role of biomarkers in iNPH diagnosis. Two studies compared biomarkers in iNPH to healthy controls, Alzheimer’s disease, and other forms of dementia [12, 48]; however, their definition of iNPH diagnosis did not require shunt response. Furthermore, these papers did not answer the most valuable question of whether a biomarker can reliably indicate if a patient will benefit from shunt insertion. Depending on the patient selection process, the reported proportion of patients responding to shunt surgery varies between 59 and 90% [24, 31, 72]. Moreover, shunt surgery in iNPH carries a significant risk of complications [41, 55, 72]. To avoid shunt surgery in iNPH patients who most likely do not respond, identifying biomarkers of shunt-responsive iNPH is highly warranted. One systematic review from 2017 [59] did explore this topic but did not include a meta-analysis. Furthermore, even the recommendations made in the latest guidelines on CSF biomarker analysis in iNPH management [56] are limited in their internal validity, as they drew their conclusion based on purely qualitative collation of different studies, without a single meta-analysis. Given these strong limitations in the current literature, our review aims to qualitatively and quantitatively evaluate the diagnostic effectiveness of the most important current biomarkers in identifying shunt-responsive iNPH, incorporating the latest primary research.

## Methods

### Literature search

This systematic review was conducted following the Cochrane Collaboration guidelines [13] and Preferred Reporting Items for Systematic Reviews and Meta-Analyses (PRISMA) [53]. Supplemental Table 1 shows the completed PRISMA Checklist. A comprehensive search of MEDLINE and Embase was conducted from January 1965 to November 2021 performed to answer the following research question: “Which cerebrospinal and venous biomarkers predict shunt-responsive iNPH?”. Normal-pressure hydrocephalus was first described in 1965 [2]. The search terms are presented in Supplementary Table 2. Additional original articles were identified by manual searching in Scopus, PubMed, Google Scholar, and JSTOR using the search strings as specified in Supplementary Table 2.

### Study inclusion and exclusion criteria

Our inclusion criteria were the following: adult iNPH patients, radiological confirmation of hydrocephalus, one or more clinical features of iNPH, use of cerebrospinal fluid shunt, objective system of functional grading of patients pre-operatively, and a minimum of 3 months post-operatively. Biochemical testing was done to predict shunt response. The exclusion criteria were the following: studies that looked solely at invasive cortical biopsies, as cortical biopsy was deemed to be not significantly less invasive than shunt insertion. In the first abstract search, all original articles in the English language that reported on iNPH diagnosis were included. Subsequently, from this preliminary list, only studies reporting on the use of biochemical markers for the prediction of shunt response in iNPH management, as well as those fulfilling our inclusion criteria, were included.

### Eligibility assessment, data extraction, and quality assessment

All included papers were assessed for eligibility independently by two independent reviewers. Any disagreements were resolved by consensus after discussion with a third and fourth reviewer. All relevant data were extracted using the Covidence data collection tool [14]. Relevant data included author names, publication dates, study type, shunted patients, study methodology (sample type, assessments, follow-up), criteria for shunt response, main reported outcomes (differences in biomarker levels in standard mean difference between shunt responders and shunt non-responders, area under curve, sensitivity, and specificity of the biomarker for predicting shunt-responsive iNPH), complications and dropout rates, funding declarations, as well as conflicts of interests. No assumptions were made regarding any studies’ content. All articles were critically appraised using the ROBINS-I tool by two independent reviewers, and a consensus was reached by discussion with a third reviewer [69]. Furthermore, the level of evidence for each included article was scored using the Oxford Centre of Evidence-Based Medicine (OCEBM) Levels of Evidence Table [34].

### Statistical analysis

An Egger’s regression and asymmetry test [13] were used to assess publication bias (*p* < 0.05% = significant). Data preparation, statistical analysis, and forest plot synthesis were carried out by utilising meta package [64] with the R software (version 4.0.4) [61]. The data sheets and R code are shown in Supplementary Tables 3–6. Stata (Release 17) was utilised to create an albatross plot [68]. A random-effects subgroup meta-analysis was conducted for each CSF biomarker that had three or more studies discussing its use. Studies must have included the following information: sample size for shunt-responsive and non-shunt-responsive group and for each biomarker, the mean, standard deviation, and *p*-value comparing the two groups. If only two studies discussed a biomarker, then the biomarker was included in the albatross plot but not in the meta-analysis. If only one study discussed a biomarker, then the biomarker was excluded from both the meta-analysis and albatross plot. Shunt responder biochemical marker data was used as the dependent variable, to which the shunt non-responder biochemical marker data was the independent variable. The inverse variance method was used for pooling effect sizes [26]. The Hartung-Knapp method was used to adjust test statistics and confidence intervals [30]. Cohen's d was utilised to estimate the standardised mean difference (SMD). The restricted maximum-likelihood estimator was used to analyse variance between studies. The *t*-test was used to calculate the overall statistical result of each meta-analysis with the associated *p*-value. Heterogeneity was estimated using the chi-squared statistic (*I*^2^) with the associated *p*-value. A statistical significance was assumed for *p* < 0.05. A sensitivity analysis was performed in two steps. Firstly, if included studies for each biomarker meta-analysis were rated at “serious” or “critical” overall risk of bias according to ROBINS-I tool, an additional subgroup random-effects meta-analysis without these studies was performed by utilising meta package [64] with the R software (version 4.0.4) [61]. Secondly, a multi-variate mixed-effects meta-regression model was built and calculated by utilising meta package [64] with the R software (version 4.0.4) [61]. The following regression equation was employed:

$${\widehat{\theta }}_{k}= \theta + {\beta }_{1}{x}_{k }+{\epsilon }_{k }+{\zeta }_{k}$$

Reading the equation left to right, ${\widehat{\theta }}_{k}$ denotes the observed effect size of each study ($k$) and acts as the dependent variable. $\theta$ denotes the y-axis intercept, and ${\beta }_{1}{x}_{k}$ is the independent variable, an arm-level covariate vector. The variables ${\epsilon }_{k }$ and ${\zeta }_{k}$ denote two independent error variables. ${\zeta }_{k}$ explains that even the measured true effect size of each study is merely sampled from an overarching effect size distribution, which implies that heterogeneity variance exists between studies. The error term ${\epsilon }_{k}$ describes the underlying independent sampling error which causes the effect size of a study to deviate from the true effect size. In this study, the following explanatory variables model was chosen to explain and represent the error term ${\epsilon }_{k}$:

$${\epsilon }_{k }=\left({{\beta }_{{2}_{age}}+\beta }_{{3}_{females}}+ {\beta }_{{4}_{sample}}+ {\beta }_{{5}_{date}}+ {\beta }_{{6}_{srm}} + {\beta }_{{7}_{neuro}}+ {\beta }_{{8}_{dropout}}\right){x}_{k}$$

The error term ${\epsilon }_{k}$ is hypothesised to be influenced by age of the patient population (${\beta }_{{2}_{age}})$, the proportion of females in the percentage of overall population sample (${\beta }_{{3}_{females}})$, the sample size (number of shunt responders and shunt non-responders) (${\beta }_{{3}_{sample}})$, the date of publication (${\beta }_{{4}_{date}})$, the method of shunt response measurement (${\beta }_{{5}_{srm}}$), and the dropout rate (${\beta }_{{9}_{dropout}}$) for each study$({x}_{k })$. The different explanatory variables were calculated singularly as sole covariates in separate meta-regressions, and if significant coefficients were yielded, further regression analyses were performed by adding additional covariates to the sole covariate to assess if significance was retained. Furthermore, a bubble plot was created using the R software (version 4.0.4) [61] to visualise the meta-regression of significant covariates. Finally, an additional meta-analysis was subsequently performed by removing the studies that caused the significant covariates. The significant studies were identified by examining the bubble plots for outliers.

## Results

**Fig. 1:**
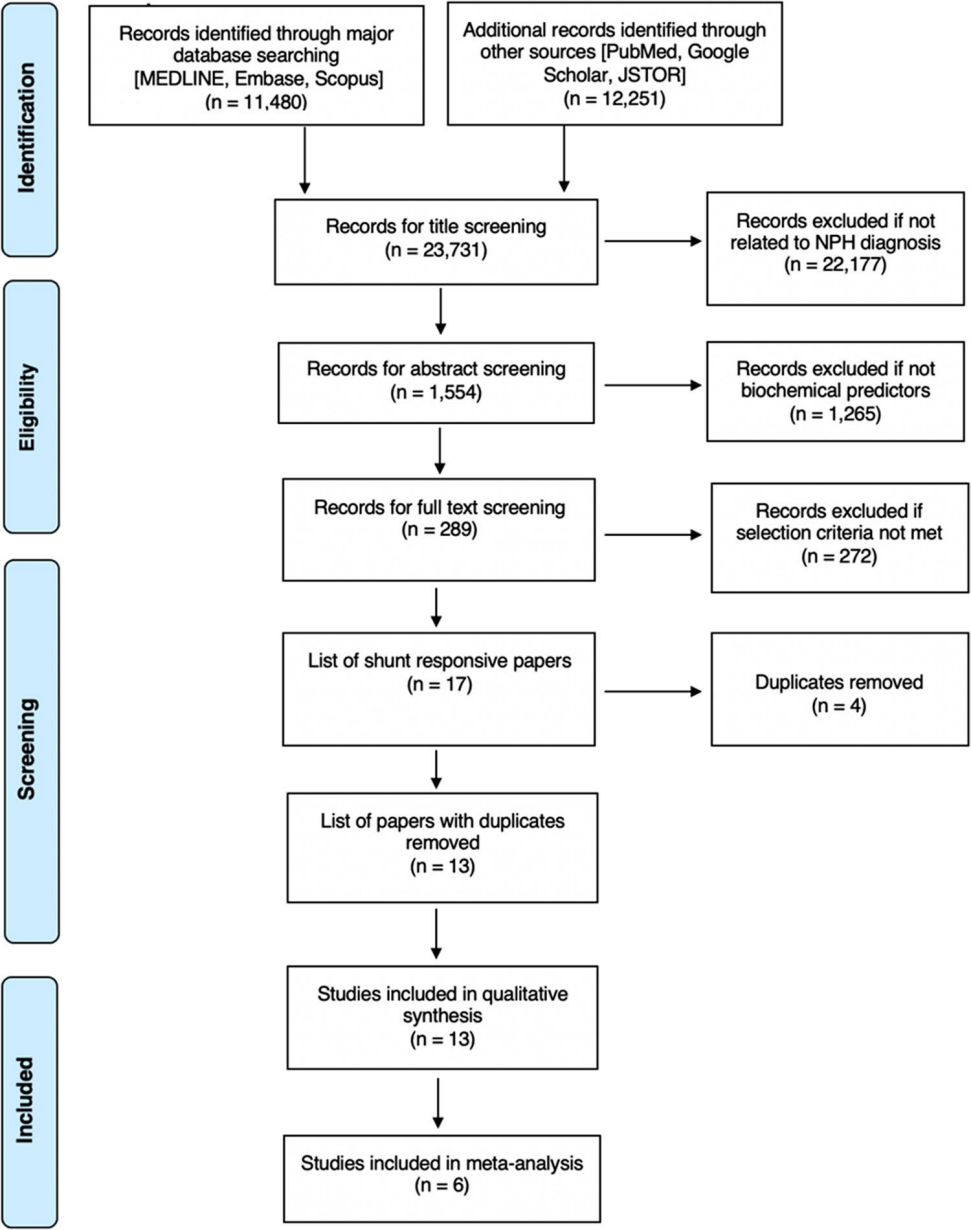
Preferred Reporting Items for Systematic Reviews and Meta-Analyses (PRISMA) flowchart outlining the study selection process

**Fig. 2:**
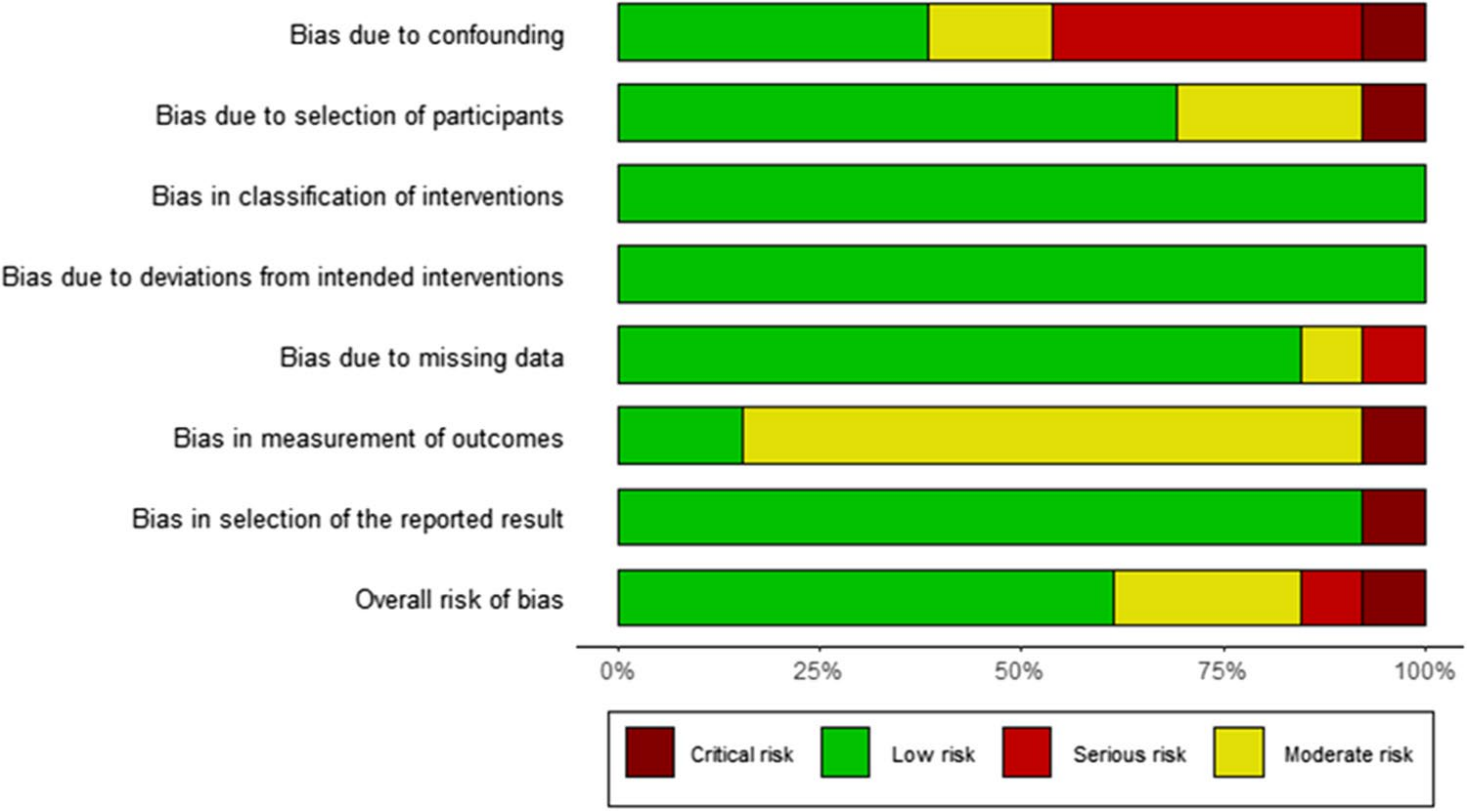
A risk of bias summary plot for non-randomised studies with bar chart of the distribution of risk-of-bias judgements for all included studies (*n* = 13) [1, 4, 15, 33, 39, 51, 52, 58, 60, 65, 70, 73, 74] across the domains of the ROBINS-I tool, shown in percentages (%) is shown. In the bottom, an overall risk of bias, which represents the collated risk-of-bias judgements for all domains, is depicted

**Fig. 3:**
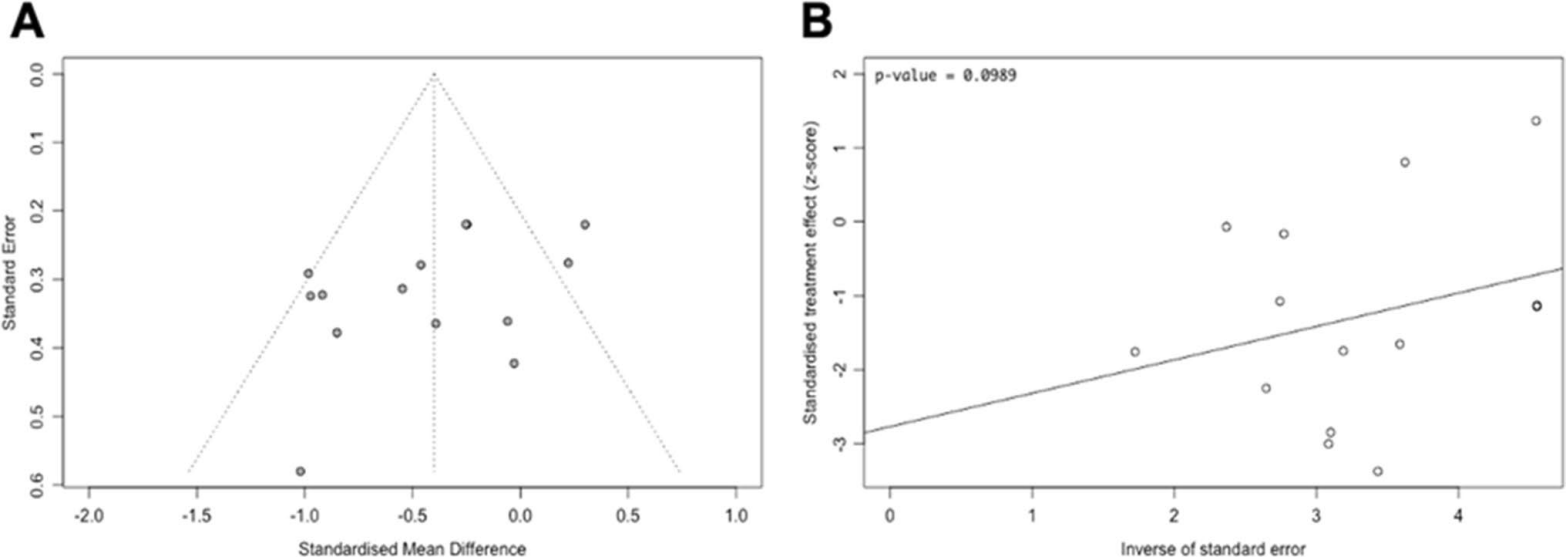
**A** A funnel plot is shown, which plots every study included in the meta-analysis (*n* = 14; 6 original studies but used and counted multiple times due to reporting on multiple biomarkers) [4, 33, 51, 70, 73, 74], particularly their observed effect sizes (standard mean difference) on the x-axis against a measure of their standard error on the y-axis. **B** An Egger’s asymmetry test funnel plot of all data points included in the meta-analysis (*n* = 14; 6 original studies but used and counted multiple times due to reporting on multiple biomarkers indicating presence and degree of publication bias is shown). *p*-value < 0.05 is deemed significant and implicates publication bias. Egger’s asymmetry test yielded *p* = 0.0989, calculated running an Egger’s regression (see Egger’s regression line) on the collated logDOR and standard errors of all data used in the meta-analysis (*n* = 14)

The literature search retrieved a total of 1,554 papers for abstract screening, of which 289 papers underwent full-text review and 13 studies were included (Fig. 1) [1, 4, 15, 33, 39, 51, 52, 58, 60, 65, 70, 73, 74]. The pooled sample size of these 13 studies was *n* = 776 shunted patients. The ROBINS-I tool scored eight of the included studies at low risk of bias overall, three studies at moderate risk, one study at serious risk, and one study at critical risk (Fig. 2). The Oxford Centre for Evidence-Based Medicine (OCEBM) analysis [34] scored 12 studies at “Level 3” [1, 4, 15, 33, 39, 51, 52, 58, 65, 70, 73, 74] and one study [60] at “Level 2”. No clear funnel plot asymmetry was detected (Fig. 3A), and similarly Egger’s test yielded no significant publication bias (*p* = 0.0989) (Fig. 3B). Thirteen studies investigated in total 21 biomarkers (Table 1, Table 2, Table 3), of which 18 were CSF biomarkers (Amyloid-β 1–42, T-Tau, P-Tau, NFL, sulfatide, albumin, vasoactive intestinal peptide (VIP), leucine-rich alpha-2 glycoprotein, and extracellular matrix proteins) and one was a genotyping biomarker (distribution of the apolipoprotein E genotype). Furthermore, two ratios of CSF biomarkers were examined for their use as biomarkers (T-Tau/amyloid-β 1–42 and P-Tau/amyloid-β 1–42) to predict shunt response in iNPH patients.

**Table 1:** The use of Amyloid-β 1–42 protein in CSF for prediction of shunt response in iNPH

Study	Type	Shunted patients	Methodology	Criteria for SR	Main reported outcomes	Complications/dropouts	Risk of bias (ROBINS-1)	Level of evidence (OCEBM)
*Migliorati *et al*.* [51]	P, NR, S, Cohort Study	*N* = 57	•**Samples analysed:** Lumbar CSF from tap test •**Assessments**: Patients with diagnosis of “probable NPH” (using clinical history, examination findings, and radiological findings) underwent a TT. They were shunted if they were a tap test responder •**Follow up:** 2 years iNPH scale score [32] compared with pre-operative iNPH scale score	•Patients who had a minimum increase of 5 points on the iNPH scale score •If increase is only in 1 domain of the iNPH score, minimum increase of 25 points in that domain	•No significant difference in pre-operative lumbar-CSF Amyloid-β 1–42 protein during TT between S-R and S-NR patients (513.3 [range 159.3–1179.1] and 793 [range 207.9–1007.6] respectively, *p* = 0.09) •Univariate logistic regression suggested amyloid-β 1–42 is associated with clinical outcome after surgery (*p* = 0.038) •Sensitivity: 72%, Specificity: 79.3%, optimal cut off 731.7 ng/L	•3 patients LTFU •2 patients died 1 year after surgery •2 patients had shunt removal after infectious complications	•A: Low risk •B: Moderate risk •C: Low risk •D: Low risk •E: Low risk •F: Moderate risk •G: Low risk •H: Moderate risk	•3
*Ågren-Wilsson *et al*.* [4]	RT, NR, S, Cohort Study	*N* = 55	•**Samples analysed**: Lumbar CSF from TT •**Assessments:** Patients with suspicion of NPH underwent MMSE, CT, or MRI, CSF constant pressure infusion test (including tap test). Gait examined before and after CSF hydrodynamic investigation •Patients with clinical diagnosis of NPH with gait disturbance as a major symptom. Urinary symptoms and cognitive decline were optional •**Follow-up:** •Routine care 3–6 months after surgery •Lumbar CSF samples collected pre-operatively and collected at 3, 9, 18, and 36 months post-operatively	Any of: •If gait speed was increased by 10% or greater •Gait score from video review was 2 or more points higher post-operatively or reached maximum points	•No difference in pre-operative amyloid-β 1–42 between S-R and S-NR patients (515 [107] and 492 [98] respectively)	•Not reported	•A: Low risk •B: Low risk •C: Low risk •D: Low risk •E: Low risk •F: Moderate risk •G: Low risk •H: Low risk	•3
*Craven *et al*.* [15]	RT. NR, S, Cohort Study	*N* = 144	**Samples analysed:** Lumbar and ventricular CSF collected during LP, ELD, or infusion study, VP shunt, VP shunt revision or reservoir tap test **Assessments:** •Wechsler Adult Intelligence Scaler R neuropsychology report (any improvement observed in verbal, performance, or full-scale IQ) •Timed 10-m walking •Bladder control **Follow-up:** Mean f/u 959 ± 657 days (mean ± SD)	Improvement in any of the following domains: •Any improvement observed in verbal, performance, or full-scale IQ •5% improvement in either walking time in seconds or number of steps, or both •Improvement in bladder control to 1 or less episodes of incontinence per day	•AUROC for lumbar CSF levels of amyloid-β 1–42 not useful prognostic tools in overall shunt response. (AUROC = 0.5) •Lumbar CSF contingency with low amyloid-β 1–42 (< 500 ng/l): Sensitivity = 79%, Specificity = 25%, PPV = 48%, NPV = 57% •Ventricular CSF levels of amyloid-β 1–42 was not significant predictor of overall shunt (*p* = 0.51) •Ventricular CSF contingency with low amyloid-β 1–42 (< 500 ng/l): Sensitivity = 64%, Specificity = 28%, PPV = 61% NPV = 32%	•6 sample error or invalid •16 LTFU •2 died before f/u	•A: Low risk •B: Low risk •C: Low risk •D: Low risk •E: Low risk •F: Low risk •G: Low risk •H: Low risk	•3
*Hong *et al*.* [33]	P, NR, M, Cohort Study	*N* = 31	•**Samples analysed**: Lumbar CSF from TT •**Assessments:** Seoul Neuropsychological Screening Battery MMSE Modified Rankin Scale (MRS) iNPH grading scale Lumbar CSF biomarkers pre-operative levels Pre-operative MRI •**Follow-up**: Routine follow-up evaluations at 1, 3, 6, and 12 months after surgery with CT at each appointment	•Patients had to show increase of 3 points or more in the iNPH grading scale or ≥ 2 points in MRS	•No difference in pre-operative amyloid-β 1–42 between S-R and S-NR patients (581.0 [173.9] and 594.3 [274.3] respectively, *p* = 0.898	•4 patients LTFU •1 patient died 1 year after surgery	•A: High risk •B: Low risk •C: Low risk •D: Low risk •E: Moderate risk •F: Moderate risk •G: Low risk •H: Moderate risk	•3
*Vanninen *et al*.* [74]	RT, NR, S, Cohort	*N* = 108	•**Samples analysed**: Lumbar CSF from TT •**Assessments:** Patients with 1 of gait difficulty, impaired cognition, urinary incontinence, and MRI showing disproportionate ratio of brain ventricles (Evans Index > 0.3) to sulci of cerebral convexities CSF (Tap Test) •**Follow-up: 3 months after surgery**	•iNPH grading scale •Clinical response at 3 months	•No significant difference tested in S-R and S-NR amyloid-β 1–42 (673.9 [192.2] and 617.5 [172.7] respectively, *p* = 0.263)	11 dropouts	•A: Low risk •B: Low risk •C: Low risk •D: Low risk •E: Low risk •F: Moderate risk •G: Low risk •H: Low risk	•3
*Tarnaris *et al*.* [70]	P, NR, S, Cohort	*N* = 22	**Samples analysed**: Ventricular CSF samples collected immediately following ventricular catheterisation **Assessments**: Black Grading Scale (BGS) **Follow-up**: 6 months	•BGS score of “Excellent”, “good”, or “fair” was determined as shunt response •BGS score of “transient”, “poor”, or “dead” was seen as a poor outcome	•Higher amyloid-β 1–42 in shunt non-responders (302.1 [96] vs 177.64 [67.9], *p* = 0.011)	•One patient LTFU	•A: Low risk •B: Low risk •C: Low risk •D: Low risk •E: Low risk •F: Moderate risk •G: Low risk •H: Low risk	•3
*Abu Hamdeh *et al*.* [1]	P, NR, S, Cohort	*N* = 20	**Samples analysed:** pre-operative lumbar CSF **Assessments:** Modified iNPH scale (Hellström et al. [32]) **Follow-up**: 3 months post-op	•Shunt response was defined as an increase of ^3^5 points at FU	•No association between CSF-Amyloid-β 1–42 (*p* = 0.09) and shunt-response	•Two patients required a shunt revision due to a catheter misplacement •Two patients with signs of overdrainage on post-operative CT scans were successfully treated by increasing the shunt valve pressure setting	•A: Low risk •B: Moderate risk •C: Low risk •D: Low risk •E: Low risk •F: Low risk •G: Low risk •H: Low risk	•3

Studies included assessing the use of any biochemical test measuring CSF Amyloid-β 1–42 protein as sole predictor of shunt responsiveness. The values presented are mean [SD], unless otherwise specified. *S-R* shunt responder, *S-NR* shunt non-responder, *amyloid-β 1–42* Beta Amyloid protein, *T-Tau* Total Tau protein, *P-Tau* Phospho-Tau protein, *LRG* Leucine-rich-alpha-2-glycoprotein, *aβO10-20* Amyloid-β oligomers, consisting of 10–20 monomers, *ECM* extracellular matrix, *MMP* matrix metalloproteinase, *TIMP-1* tissue inhibitor matrix metalloproteinase 1, *VIP* vasoactive intestinal polypeptide, *TT* tap test, *MMSE* Mini Mental State Examination, *LP* lumbar puncture, *ELD* external lumbar drainage, *MRS* Modified Rankin Scale, *BGS* Black Grading Scale, *LTFU* lost-to-follow-up, *SMD* Standard Mean Difference.

Study type: *P* prospective, *RT* retrospective, *R* randomised, *NR* non-randomised, *M* multi-centre, *S* single-centre.

ROBINS-I analysis [69]: A, bias due to confounding; B, bias in selection of participants into the study; C, bias in classification of interventions; D, bias due to deviations from intended interventions; E, bias due to missing data; F, bias in measurement of outcomes; G, bias in selection of the reported result; H, Overall bias; Oxford Centre for Evidence-Based Medicine (OCEBM) analysis [34]: Levels 1–5.

**Table 2:** The use of Tau proteins in CSF for prediction of shunt response in iNPH

Study	Type	Shunted patients	Methodology	Criteria for SR	Main reported outcomes	Complications/dropouts	Risk of bias (ROBINS-1)	Level of evidence (OCEBM)
*Migliorati *et al*.* [51]	P, NR, S, Cohort Study	*N* = 57	•**Samples analysed:** Lumbar CSF from TT •**Assessments**: Patients with diagnosis of “probable NPH” (using clinical history, examination findings, and radiological findings) underwent a tap test. They were shunted if they were a TT responder •**Follow up:** 2 years iNPH scale score [32] compared with pre-operative iNPH scale score	•Patients who had a minimum increase of 5 points on the iNPH scale score •If increase only in 1 domain of the iNPH score, increase ≥ 25 points in said domain = SR	•Significant difference in pre-operative lumbar-CSF T-Tau protein levels during TT between S-R and S-NR patients (161.1 [range 37.5–779.9] and 245 [range 118.5–670.4] respectively, *p* = 0.02) •Univariate logistic regression suggested T-Tau levels 233.9 ng/l are significantly associated with clinical outcome after surgery (*p* = 0.024) -Sensitivity: 81.8%, specificity 72.4%, for a cut-off at 233.9 ng/L •Significant difference in pre-operative lumbar-CSF P-Tau protein during TT between S-R and S-NR patients (26.6 [range 15.5–70.5] and 39.6 [range 21.2–78.5] respectively, *p* = 0.01) •Univariate logistic regression suggested p-Tau levels 233.9 ng/l are significantly associated with clinical outcome after surgery (*p* = 0.009) -Sensitivity: 81.8%, Specificity 72.4%, For A Cut-off at 32.2 ng/L	•3 patients LTFU •2 patients died 1 year after surgery •2 patients had shunt removal after infectious complications	•A: Low risk •B: Moderate risk •C: Low risk •D: Low risk •E: Low risk •F: Moderate risk •G: Low risk •H: Moderate risk	•3
*Ågren-Wilsson *et al*.* [4]	RT, NR, S, Cohort Study	*N* = 55	•**Samples analysed**: Lumbar CSF from TT •**Assessments:** Patients with suspicion of NPH underwent MMSE, CT or MRI, CSF constant pressure infusion test (including tap test). Gait examined before and after CSF hydrodynamic investigation •Patients with clinical diagnosis of NPH with gait disturbance as a major symptom. Urinary symptoms and cognitive decline were optional •**Follow-up:** •Routine care 3–6 months after surgery •Lumbar CSF samples collected pre-operatively and collected at 3, 9, 18, and 36 months post-operatively	Any of: •If gait speed was increased by 10% or greater •Gait score from video review was 2 or more points higher post-operatively or reached maximum points	•No difference in pre-operative T-Tau between S-R and S-NR patients (164 [65] and 180 [62] respectively) •No difference in pre-operative P-Tau between S-R and S-NR patients (32 [10] and 37 [12] respectively	•Not reported	•A: Low risk •B: Low risk •C: Low risk •D: Low risk •E: Low risk •F: Moderate risk •G: Low risk •H: Low risk	•3
*Craven *et al*.* [15]	RT. NR, S, Cohort	*N* = 144	**Samples analysed:** Lumbar and ventricular CSF collected during LP, ELD or infusion study, VP shunt, VP shunt revision, or reservoir tap test **Assessments:** •Wechsler Adult Intelligence Scaler R neuropsychology report (any improvement observed in verbal, performance, or full-scale IQ) •Timed 10-m walking •Bladder control **Follow-up:** Mean f/u 959 ± 657 days (mean ± SD)	Improvement in any of the following domains: •any improvement observed in verbal, performance, or full-scale IQ •5% improvement in either walking time in seconds or number of steps, or both •Improvement in bladder control to 1 or less episodes of incontinence per day	•AUROC for lumbar CSF levels of T-tau and T-Tau/ amyloid-β 1–42 not useful prognostic tools in overall shunt response. (AUROC respectively 0.6, 0.62) •Ventricular CSF levels of T-Tau and T-Tau/ amyloid-β 1–42 were not significant predictors of overall shunt *p* = 0.70 and *p* = 0.64, respectively •Lumbar CSF T-tau significant (AUROC 0.84, *p* = 0.04) as predictor of shunt response in terms of mobility	•6 sample error or invalid •16 lost to f/u •2 died before f/u	•A: Low risk •B: Low risk •C: Low risk •D: Low risk •E: Low risk •F: Low risk •G: Low risk •H: Low risk	•3
*Hong *et al*.* [33]	P, NR, M, Cohort Study	*N* = 31	•**Samples analysed**: Lumbar CSF from TT •**Assessments:** Seoul Neuropsychological Screening Battery MMSE Modified Rankin scale (MRS) iNPH grading scale Lumbar CSF biomarkers pre-operative levels Pre-operative MRI •**Follow-up**: Routine follow-up evaluations at 1, 3, 6, and 12 months after surgery with CT at each appointment	•Patients had to show increased of 3 or more in iNPH grading scale or 2 or more in MRS	•No significant difference in pre-operative T-Tau between S-R and S-NR patients (141.9 [40.8] and 177.2 [126.7] respectively, *p* = 0.410) •No significant difference in pre-operative P-Tau between S-R and S-NR patients (29.8 (12.9) and 47.6 (27.8) respectively, *p* = 0.078) •Significant difference in pre-operative P-Tau/amyloid-β 1–42 ratio between S-R and S-NR patients (0.06 [0.03] and 0.10 [0.07] respectively, *p* = 0.041) •No significant difference in pre-operative T-Tau/amyloid-β 1–42 ratio between S-R and S-NR patients (0.29 [0.18] and 0.34 [0.26], respectively, *p* = 0.564)	•4 patients LTFU •1 patient died 1 year after surgery	•A: High risk •B: Low risk •C: Low risk •D: Low risk •E: Moderate risk •F: Moderate risk •G: Low risk •H: Moderate risk	•3
*Vanninen *et al*.* [74]	RT, NR, S, Cohort	*N* = 108	•**Samples analysed**: Lumbar CSF from TT •**Assessments:** Patients with 1 of gait difficulty, impaired cognition, urinary incontinence, and MRI showing disproportionate ratio of brain ventricles (Evans Index > 0.3) to sulci of cerebral convexities CSF (Tap Test) •**Follow-up: 3 months after surgery**	•iNPH grading scale •Clinical response at 3 months	•No significant difference in S-R and S-NR •P-Tau (34.7 [14.8] and 38.3 [12.2] respectively, *p* = 0.683) •T-Tau (190.4 [86.8] and 211.3 [75.9] respectively, *p* = 0.822)	11 dropouts	•A: Low risk •B: Low risk •C: Low risk •D: Low risk •E: Low risk •F: Moderate risk •G: Low risk •H: Low risk	•3
*Patel *et al. [58]	P, NR, S, Cohort	*N* = 39	**Samples analysed:** Ventricular CSF obtained intraoperatively. A subset of 18 patients also had lumbar CSF sampling **Assessments:** 15-point NPH scale (three five-point subscale describing deficits in gait, cognition, and bladder control) Cognition was evaluated using a psychometric battery comprised of the WMS-III logical memory subtest, a 10-item word list recall task, digit span forward and reversed, category fluency, Boston Naming test, trial making, digit symbol and clock draw and copy **Follow-up: 4** months post-op	Paired t-tests comparison of pre-vs post-shunting clinical assessment score A one-sample t-test to evaluate percentage change in gait time	•Significantly lower (*t* = 2.26, *df* = 27, *p* = 0.032) percent change in gait time in the group with higher P-Tau/amyloid-β 1–42 (− 8.3 [ 33.4]) compared to the group with lower P-Tau/Aβ-42 (− 35.1 [28.3])	Not reported	•A: Moderate risk •B: Moderate risk •C: Low risk •D: Low risk •E: High risk •F: Critical risk •G: Low risk •H: Serious risk	•3
*Tarnaris *et al*.* [70]	P, NR, S, Cohort	*N* = 22	**Samples analysed**: Ventricular CSF samples collected immediately following ventricular catheterisation **Assessment**s: Black grading scale (BGS) **Follow-up**: 6 months	•BGS score of “Excellent”, “Good”, or “Fair” was determined as shunt response •BGS score of “transient,” “poor” or “dead” was seen as a poor outcome	•S-NR showed increased T-Tau (1086.23 [347.19] vs 550.97 [551.65] in S-R, *p* = 0.025)	•One patient LTFU	•A: Low risk •B: Low risk •C: Low risk •D: Low risk •E: Low risk •F: Moderate risk •G: Low risk •H: Low risk	•3
*Tullberg *et al*.* [73]	P, NR, S, Cohort	*N* = 35	**Sample analysed:** Pre-operative lumbar CSF from LP **Assessments:** Global: •MMSE (0–30) Psychometric: •Identical forms test (0–60) •Bingley’s visual recognition test (0–12) •Reaction time test (exact time in seconds) Balance •Romberg’s test (0–60) •Gait (1–6) •Walking 10 m (time and steps needed respectively) Continence •Urgency incontinence (1–2, where 1 = not present and 2 = present) •**Follow-up**: 3-months post-op	Pre- and post-operative values for each index were calculated and a mean of the differences (MoD) calculated as the overall result of the surgery •Patients with a MoD > 0.05 were considered improved	•No significant difference between S-R and S-NR T-Tau (267 [296] vs 275 [165])	•None reported	•A: Critical risk •B: Low risk •C: Low risk •D: Low risk •E: Low risk •F: Moderate risk •G: Critical risk •H: Critical risk	•3
*Abu Hamdeh *et al*.* [1]	P, NR, S, Cohort	*N* = 20	**Samples analysed:** pre-operative Lumbar CSF **Assessments:** Modified iNPH scale (Hellström et al., [32]) **Follow-up**: 3 months post-op	•Shunt response was defined as an increase of ^3^5 points at FU	•No association between CSF Amyloid-β 1–42 (*p* = 0.09), CSF-T-Tau (*p* = 0.21), or CSF P-Tau (*p* = 0.17) and shunt response	•Two patients required a shunt revision due to a catheter misplacement •Two patients with signs of over drainage on post-operative CT scans were successfully treated by increasing the shunt valve pressure setting	•A: Low risk •B: Moderate risk •C: Low risk •D: Low risk •E: Low risk •F: Low risk •G: Low risk •H: Low risk	•3

Studies included assessing the use of any biochemical test measuring CSF Tau proteins as predictor of shunt responsiveness, as sole predictor and in a ratio with other CSF proteins. The values presented are mean [SD], unless otherwise specified. *S-R* shunt responder, *S-NR* shunt non-responder, *amyloid-β 1–42* Beta Amyloid protein, *T-Tau* Total Tau protein, *P-Tau* Phospho-Tau protein, *LRG* Leucine-rich-alpha-2-glycoprotein, *aβO10-20* Amyloid-β oligomers, consisting of 10–20 monomers, *ECM* extracellular matrix, *MMP* matrix metalloproteinase, *TIMP-1* tissue inhibitor matrix metalloproteinase 1, *VIP* vasoactive intestinal polypeptide, *TT* Tap test, *MMSE* Mini Mental State Examination, *LP* lumbar puncture, *ELD* external lumbar drainage, *MRS* Modified Rankin Scale, *BGS* Black Grading Scale, *LTFU* lost-to-follow-up, *SMD* standard mean difference.

Study type: *P* prospective, *RT* retrospective, *R* randomised, *NR* non-randomised, *M* multi-centre, *S* single-centre.

ROBINS-I analysis [69]: A, bias due to confounding; B, bias in selection of participants into the study; C, bias in classification of interventions; D, bias due to deviations from intended interventions; E, bias due to missing data; F, bias in measurement of outcomes; G, bias in selection of the reported result; H, Overall bias; Oxford Centre for Evidence-Based Medicine (OCEBM) analysis [34]: Levels 1–5.

**Table 3:** The use of miscellaneous biomarkers (CSF proteins, ECM proteins, proteomics, and genotyping) for prediction of shunt response in iNPH

Study	Type	Shunted patients	Methodology	Criteria for SR	Main reported outcomes	Complications/dropouts	Risk of bias (ROBINS-1)	Level of evidence (OCEBM)
*Ågren-Wilsson *et al*.* [4]	RT, NR, S, Cohort Study	*N* = 55	•**Samples analysed**: Lumbar CSF from TT •**Assessments:** Patients with suspicion of NPH underwent MMSE, CT or MRI, CSF constant pressure infusion test (including tap test). Gait examined before and after CSF hydrodynamic investigation •Patients with clinical diagnosis of NPH with gait disturbance as a major symptom. Urinary symptoms and cognitive decline were optional •**Follow-up:** •Routine care 3–6 months after surgery •Lumbar CSF samples collected pre-operatively and collected at 3, 9, 18, and 36 months post-operatively	Any of: •If gait speed was increased by 10% or greater •Gait score from video review was 2 or more points higher post-operatively or reached maximum points	•No difference in pre-operative NFL between S-R and S-NR patients (680 [666] and 1008 [1118], respectively) •No difference in pre-operative Sulfatide between S-R and S-NR patients (275 [115] and 269 [93], respectively)	•Not reported	•A: Low risk •B: Low risk •C: Low risk •D: Low risk •E: Low risk •F: Moderate risk •G: Low risk •H: Low risk	•3
*Vanninen *et al*.* [74]	RT, NR, S, Cohort	*N* = 108	•**Samples analysed**: Lumbar CSF from TT •**Assessments:** Patients with 1 of gait difficulty, impaired cognition, urinary incontinence, and MRI showing disproportionate ratio of brain ventricles (Evans Index > 0.3) to sulci of cerebral convexities CSF (Tap Test) •**Follow-up: 3 months after surgery**	•iNPH grading scale •Clinical response at 3 months	•No significant difference in S-R and S-NR •LRG (599.0 [445.1] and 652.7 [439.0] respectively, *p* = 0.636)	11 dropouts	•A: Low risk •B: Low risk •C: Low risk •D: Low risk •E: Low risk •F: Moderate risk •G: Low risk •H: Low risk	•3
*Pyykkö *et al. [60]	R, NR, case–control	*N* = 202	•**Sample analysed:** Venous blood •**Assessments:** Patients were selected from the local NPH registry if fulfilling the following criteria: (1) primary evaluation and examination by a neurologist; (2) one to three symptoms suggestive of NPH (gait disorder, cognitive impairment, and urinary incontinence); and (3) NPH related brain imaging findings •**Follow-up**: patients were followed up until death or the end of Nov 2010. Median FU 3.9 years (range 0.2–17.3 yrs)	Any subjective or objective improvement in patient gait, memory or urinary continence was graded as a positive shunt response	•The 94 shunt-responsive and 16 non-responsive iNPH patients had a similar distribution of APoE genotypes (*p* = 0.47) and proportion of APoE4 carriers (19% vs 19%; *p* = 0.72)	•3 patent insufficient FU data for a final clinical diagnosis of Alzheimer’s dementia	•A: High risk •B: Critical risk •C: Low risk •D: Low risk •E: Low risk •F: Moderate risk •G: Low risk •H: Low risk	•2
*Scollato *et al. [65]	P, NR, S, Cohort	*N* = 17	•**Sample analysed:** Intraoperative collection of Ventricular CSF during catheter insertion •**Assessments:** Clinical score comprised of: •MMSE (0–30) •Gait scale (GS, 0–4) •Urinary incontinence scale (UIS, 0–3) •**Follow-up:** 6 months post-op	•Comparison of clinical score before and after shunting (denoted by Δ) to form a clinical evolution score (CES) •CES = ΔUIS + ΔGS + ΔMMSE/3 •CES ≥ 3 indicated shunt response	In S-NR the following was observed: •Increased expression of Clusterin, Apo J, Apo E, GFAP •Decreased expression of a2-HS-GP, a1b-GP	•None reported	•A: High risk •B: Low risk •C: Low risk •D: Low risk •E: Low risk •F: Moderate risk •G: Low risk •H: Moderate risk	•3
*Tullberg *et al. [73]	P, NR, S, Cohort	*N* = 35	**Sample analysed:** Pre-operative lumbar CSF from LP **Assessments:** Global: •MMSE (0–30) Psychometric: •Identical forms test (0–60) •Bingley’s visual recognition test (0–12) •Reaction time test (exact time in seconds) Balance •Romberg’s test (0–60) •Gait (1–6) •Walking 10 m (time and steps needed respectively) Continence •Urgency incontinence (1–2, where 1 = not present and 2 = present) •**Follow-up**: 3 months post-op	Pre- and post-operative values for each index were calculated and a mean of the differences (MoD) calculated as the overall result of the surgery Patients with a MoD > 0.05 were considered improved	No significant difference between S-R and S-NR CSF concentration in the following parameters •Total albumin (650 [413] vs 529 [211]), albumin ratio (10.8 [8.9] vs 8.0 [4.6]), respectively •HVA (240 [154] vs 140 [102]), 5-HIAA (126 [80] vs 74 [53]), HMPG (39 [16] vs 28 [13]), NPY (95 [30] vs 108 [39]), VIP (6.4 [1.4] vs 10.5 [7.9]), GABA (53 [25] vs 80 [58]) •Sulphatide (210 [100] vs 204 [105]) •GD3 (35 [22] vs 50 [25]) •NFL (3637 [5338] vs 938 [1102]), T-Tau (267 [296] vs 275 [165])	None reported	•A: Critical risk •B: Low risk •C: Low risk •D: Low risk •E: Low risk •F: Moderate risk •G: Critical risk •H: Critical risk	•3
*Johansson *et al*.* [39]	P, NR, Cohort Study, *	*N* = 18	•**Sample Analysed:** lumbar CSF •**Assessments:** NPH diagnosed by characteristic neurological symptoms – gait abnormalities, dementia, and urinary incontinence + characteristic changes on CT and radionuclide cisternography **Follow-up:** Second CSF sample obtained after 3 months. Third CSF sample obtained after 12 months	•Not stated	Pre-operative levels of VIP < 20 pmol/L were S-R (*N* = 11 out of 15 patients, unknown statistical significance) All patients with pre-operative levels of VIP > 20 pmol/L were S-NR (*N* = 3, unknown significance)	•None reported	•A: Moderate risk •B: Low risk •C: Low risk •D: Low risk •E: Low risk •F: Moderate risk •G: Low risk •H: Low risk	•3
*Minta *et al*.* [52]	P, NR, S, Cohort Study	*N* = 28	•**Sample Analysed:** Pre-operative ventricular and lumbar CSF •**Assessments:** NPH diagnosed using international criteria •Severity classified by iNPH scale by Hellstrom et al. [32] assessing gait, balance, cognition, and continence •**Follow-up:** 4 months post-operatively	•Post-operative iNPH scale improved by > = 5 points	•No difference in ECM proteins between S-R (*n* = 20) and S-NR (*n* = 8) pre-operatively (no significance values given)	•3 patients had complications due to another condition (multiple sclerosis, amyotrophic lateral sclerosis or disseminated malignant disease)	•A: Low risk •B: Low risk •C: Low risk •D: Low risk •E: Low risk •F: Moderate risk •G: Low risk H: Low risk	•3

Studies included assessing the use of any biochemical test using miscellaneous CSF proteins (excluding amyloid-β 1–42 or Tau proteins), proteomics, and genotyping in predicting shunt responsiveness. The values presented are mean [SD], unless otherwise specified. *S-R* shunt responder, *S-NR* shunt non-responder, *amyloid-β 1–42* Beta Amyloid protein, *T-Tau* Total Tau protein, *P-Tau* Phospho-Tau protein, *LRG* Leucine-rich-alpha-2-glycoprotein, *aβO10-20* Amyloid-β oligomers, consisting of 10–20 monomers, *ECM* extracellular matrix, *NFL* neurofilament triplet protein, *MMP* matrix metalloproteinase, *TIMP-1* tissue inhibitor matrix metalloproteinase 1, *VIP* vasoactive intestinal polypeptide, *HVA* homovanillic acid, *5-HIAA* 5-hydroxy-indoleacetic acid, *HMPG* 4-Hydroxy-3-Methoxyphenylglycol, *NPY* neuropeptide Y, *GABA* Gamma amino butyric acid, *a2-HS-GP* Alpha 2 Heremans–Schmid glycoprotein, *a1b-GP* Alpha 1 beta glycoprotein, *GFAP* glial fibrillary acid, *TT* tap test, *MMSE* Mini Mental State Examination, *LP* lumbar puncture, *ELD* external lumbar drainage, *MRS* Modified Rankin Scale, *BGS* Black Grading Scale, *FU* follow-up, *LTFU* lost-to-follow-up, *SMD* standard mean difference.

*Unknown if multi-centre or single-centre.

Study type: *P* prospective, *RT* retrospective, *R* randomised, *NR* non-randomised, *M* multi-centre, *S* single-centre.

ROBINS-I analysis [69]: A, bias due to confounding; B, bias in selection of participants into the study; C, bias in classification of interventions; D, bias due to deviations from intended interventions; E, bias due to missing data; F, bias in measurement of outcomes; G, bias in selection of the reported result; H, Overall bias; Oxford Centre for Evidence-Based Medicine (OCEBM) analysis [34]: Levels 1–5.

### Amyloid-β 1–42

Seven studies investigated lumbar CSF amyloid-β 1–42 as a prognostic biomarker in predicting shunt response (Table 1). Of these, Tarnaris et al. (2011) [70] reported higher pre-operative amyloid-β 1–42 lumbar CSF levels in shunt non-responders (*p* = 0.011). In contrast, the remainder of the studies reported no significant difference in the CSF levels of amyloid-β 1–42 between shunt responders and shunt non-responders [1, 4, 15, 33, 51, 74]. Craven et al. (2017) [15] explored the pre-operative level of amyloid-β 1–42 in ventricular CSF in shunt responders and shunt non-responders, which was insignificant (*p* = 0.51). At a cut-off level 500 ng/l, Craven et al. (2017) [15] reported a sensitivity of 79% and a specificity of 25%, but their area under the receiving operating characteristic (AUROC) analysis was insignificant at 0.5. However, Migliorati et al. (2020) [51] performed a univariate logistic regression showing that lumbar CSF levels of amyloid-β 1–42 exceeding 731.7 ng/l were significantly associated with poor shunt response (*p* = 0.038). The best cut-off identified was, after receiver operating characteristic (ROC) analysis, set at 731.7 ng/L for lumbar CSF amyloid-β 1–42 levels, yielding a sensitivity of 72.7% and a specificity of 79.3% for predicting shunt response.

### Total-Tau

Nine studies investigated the use of T-Tau to predict shunt response (Table 2) [1, 4, 15, 33, 51, 58, 70, 73, 74]. Of these, three studies reported that T-Tau was a prognostic biomarker that displayed a significant difference between shunt-responsive and non-responsive patients when comparing the pre-operative lumbar levels of T-Tau. Craven et al. (2017) [15] and Migliorati et al. (2020) [51] demonstrated that this significant difference was in lumbar CSF levels of T-Tau (*p* = 0.04 and *p* = 0.02, respectively), whereas Tarnaris et al. (2011) [70] demonstrated a significant difference in ventricular CSF levels of T-Tau. All three studies [15, 51, 70] found that the levels of T-Tau were higher in shunt-non-responsive patients. On the other hand, six studies reported no differences between levels of T-Tau in patients who were shunt-responsive and those who were shunt-non-responsive. Abu Hamdeh et al. (2018) [1], Ågren-Wilsson et al. (2007) [4], Hong et al. (2018) [33], Tullberg et al. (2008) [73], and Vanninen et al. (2021) [74] found no differences in the levels of T-Tau in lumbar CSF between shunt responders and shunt non-responders, and Craven et al. (2017) [15] found no differences in the levels of ventricular T-Tau between shunt-responsive and shunt-non-responsive patients. However, Migliorati et al. (2020) [51] performed a univariate logistic regression showing that lumbar CSF levels of T-Tau were significantly associated with the worse clinical outcomes following shunt surgery if lumbar CSF T-Tau levels exceeding 731.7 ng/l (*p* = 0.024). The best cut-off identified by ROC analysis was at the level of 233.9 ng/L, with a sensitivity of 81.8% and a specificity of 72.4% for predicting shunt response.

### Phosphorylated-Tau

Five studies analysed the use of P-Tau to predict shunt responders (Table 2) [1, 4, 33, 51, 74]. Migliorati et al. (2020) [51] reported a significantly higher lumbar CSF level of P-Tau in shunt non-responsive patients (*p* = 0.01). This finding was not reciprocated by four other studies, which showed no significant prognostic value in lumbar P-Tau [1, 4, 33, 74]. Migliorati et al. (2020) [51] also performed a univariate logistic regression for P-Tau, demonstrating that lumbar CSF levels of P-Tau exceeding 32.2 ng/L were significantly associated with poor shunt response (*p* = 0.009). The best cut-off identified was at the level of 32.2 ng/L, with a sensitivity of 81.8% and a specificity of 72.4%.

### Phosphorylated-Tau/amyloid-β 1–42 ratio

Two studies examined the difference in pre-operative P-Tau/amyloid-β 1–42 ratio between shunt-responsive and shunt-non-responsive patients (Table 2). Both studies by Hong et al. (2018) [33] and Patel et al. (2012) [58] found that there was a significantly lower ratio in patients who were shunt-responsive versus those who were shunt-non-responsive (*p* = 0.041 and *p* = 0.032, respectively).

### Total-Tau/amyloid-β 1–42 ratio

Two studies explored the difference in pre-operative T-Tau/amyloid-β 1–42 ratio between shunt-responsive and shunt-non-responsive patients (Table 2). Both studies by Craven et al. (2017) [15] and Hong et al. (2018) [33] found that there was no significant difference in the ratio between patients who were shunt-responsive versus those who were shunt-non-responsive (*p* = 0.64, and *p* = 0.564, respectively).

### Neurofilament light protein

The axonal integrity biomarker NFL protein, found in lumbar CSF, was analysed, for how its levels in the CSF are related to shunt response by two studies [4, 73] (Table 3). Both reported no significant difference in post-shunt outcomes in pre-operative NFL between shunt-responsive and shunt-non-responsive patients (Ågren-Wilsson et al. (2007) [4], *p* = 0.18; Tullberg et al. (2008) [73], *p* > 0.05).

### Albumin

One study, by Tullberg et al. (2008) [73], explored the relationship between levels of albumin and shunt outcomes (Table 3). The authors found that there was no significant difference in pre-operative levels of total albumin or the CSF/serum albumin ratio (*p* values not reported) between shunt-responsive and shunt-non-responsive patients.

### Vasoactive intestinal peptide

Differences in VIP, a neuropeptide released by immune cells and neurons found widely in the central nervous system [17], were examined by two studies with mixed results for its use to predict shunt response (Table 3). Tullberg et al. (2008) [73] reported no significant difference in pre-operative lumbar CSF VIP concentration between shunt-responsive and shunt-non-responsive patients (*p*-value not reported). In contrast, Johansson et al. [39] reported that pre-operative levels of VIP < 20 pmol/L were predictive of positive shunt response; however, the authors failed to delineate this claim with the data presented in their paper.

### Sulfatide

Sulfatide is a component of the myelin sheath in the central and peripheral nervous systems [18]. Two studies, Ågren-Wilsson et al. (2007) [4] and Tullberg et al. (2008) [73], explored the levels of sulfatide and shunt outcomes (Table 3). However, both studies reported that the differences in levels of sulfatide between patients who were shunt-responsive and those that were shunt-non-responsive were insignificant. Both studies did not report *p*-values, but the value for Ågren-Wilsson et al. (2007) [4] was calculated to be 0.84.

### Leucine-rich alpha-2 glycoprotein

Vanninen et al. (2021) [74] examined the correlation between leucine-rich alpha-2 glycoprotein (LRG) in iNPH patients undergoing shunt surgery (Table 3). LRG is a novel biomarker that is indicative of inflammation, especially autoimmune conditions [66]. The authors reported that although LRG levels are raised in iNPH, this protein is not predictive of shunt response (*p* = 0.636).

### ECM proteins

Minta et al. (2021) [52] looked at the differences in levels of extracellular matrix proteins (Brevican, Neurocan, matrix metalloproteinases (MMP)) and tissue inhibitor matrix metalloproteinase 1 (TIMP-1) between shunt-responsive and shunt-non-responsive patients (Table 3). They found that there were no significant differences in the levels of Brevican, Neurocan, MMP, or TIMP-1.

### Proteomics

Scollato et al. [65] explored proteomic differences of ventricular CSF in shunt-responsive and shunt-non-responsive patients through the means of 2D-Gel electrophoresis and matrix-assisted laser desorption/ionisation and time of flight mass spectrometry (MALDI TOF MS) (Table 3). Shunt-non-responsive patients’ samples were found to have increased expression of Clusterin, Apo J, Apo E, and GFAP, whereas a2-HS-GP and a1b-GP expression was reduced in shunt-non-responsive patients.

### Genotyping proteins

One study by Pyykkö et al. [60] looked at the distribution of the Apolipoprotein E (ApoE) genotype among patients with iNPH, specifically looking at the differences in the proportion of the ApoE4 genotype by analysing a venous blood sample (Table 3). In the population studied, there was no difference in the distribution of ApoE genotypes (*p* = 0.47), nor in the proportion of ApoE4 carriers (*p* = 0.72).

## Statistical results

### Meta-analysis

**Fig. 4:**
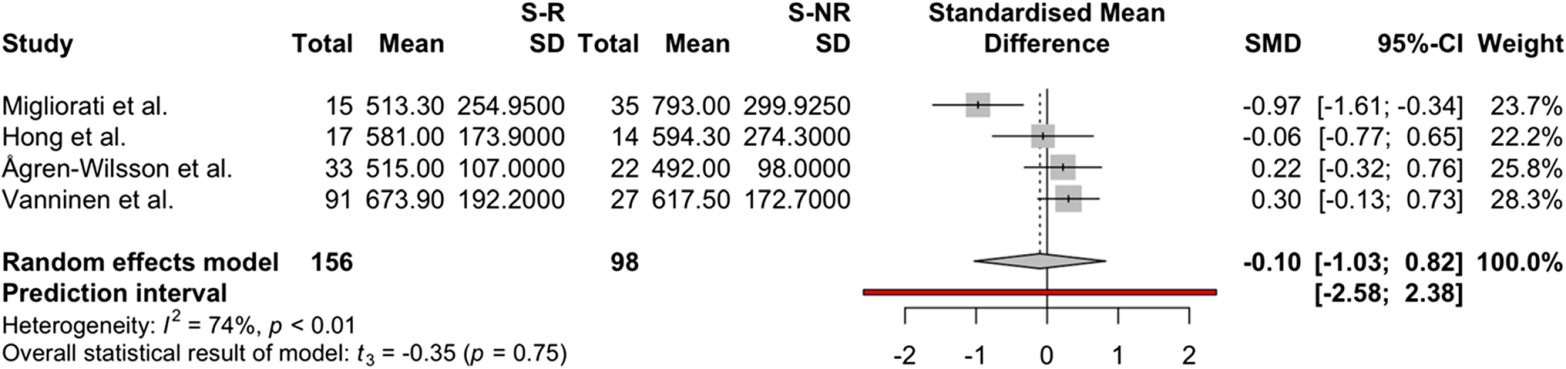
A forest plot indicating and visualising the effect size in standard mean difference (SMD) of amyloid-β 1–42 levels in lumbar CSF samples of shunt responder (S-R) versus shunt non-responder (S-NR) iNPH patients is shown (*n* = 4 studies) [4, 33, 51, 74]. The size of the grey square of the SMD visual correlates to study sample size, and the straight line indicated the confidence interval. The diamond at the bottom indicates the overall pooled effect. The red bar below it indicates the prediction interval. Heterogeneity is indicated by the chi-squared statistic (*I*^2^) with associated *p*-value. The 95% confidence intervals (CI) are shown in squared bracket ([]). Furthermore, for every study, the following are displayed: author, total number of S-R and their respective mean level and standard deviation (SD) of amyloid-β 1–42 lumbar CSF levels, as well as the respective values for S-NR, weighting of each study in percentage (%). There was no significant difference in amyloid-β 1–42 between S-R and S-NR groups

**Fig. 5:**
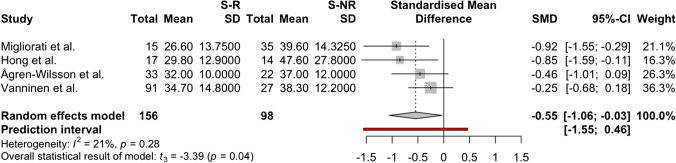
A forest plot indicating and visualising the effect size in standard mean difference (SMD) of Phosphorylated-Tau (P-Tau) levels in lumbar CSF samples of shunt responder (S-R) versus shunt non-responder (S-NR) iNPH patients is shown (*n* = 4 studies) [4, 33, 51, 74]. The size of the grey square of the SMD visual correlates to study sample size, and the straight line indicated the confidence interval. The diamond at the bottom indicates the overall pooled effect. The red bar below it indicates the prediction interval. Heterogeneity is indicated by the chi-squared statistic (*I*^2^) with associated *p*-value. The 95% confidence intervals (CI) are shown in squared bracket ([]). Furthermore, for every study, the following are displayed: author, total number of S-R and their respective mean level and standard deviation (SD) of P-Tau lumbar CSF levels, as well as the respective values for S-NR, weighting of each study in percentage (%). There was a significantly higher level of P-Tau in the S-NR group compared to the S-R group

**Fig. 6:**
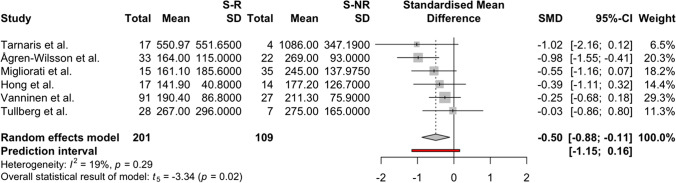
A forest plot indicating and visualising the effect size in standard mean difference (SMD) of Total-Tau (T-Tau) levels in lumbar (*n* = 5) [4, 33, 51, 73, 74] and ventricular (*n* = 1, Tarnaris et al. (2011) [70] samples of shunt responder (S-R) versus shunt non-responder (S-NR) iNPH patients is shown (*n* = 6 studies) [4, 33, 51, 70, 73, 74]. The size of the grey square of the SMD visual correlates to study sample size, and the straight line indicated the confidence interval. The diamond at the bottom indicates the overall pooled effect. The red bar below it indicates the prediction interval. Heterogeneity is indicated by the chi-squared statistic (*I*^2^) with associated *p*-value. The 95% confidence intervals (CI) are shown in squared bracket ([]). Furthermore, for every study, the following are displayed: author, total number of S-R, and their respective mean level and standard deviation (SD) of T-Tau lumbar CSF levels, as well as the respective values for S-NR, weighting of each study in percentage. There was a significantly higher level of T-Tau in the S-NR group compared to the S-R group

The meta-analysis was conducted for the following CSF biomarkers, which met the inclusion criteria for meta-analysis (studies per biomarker: *n* > 2): amyloid-β 1–42, P-Tau, and T-Tau. For amyloid-β 1–42, four studies [4, 33, 51, 74], two scoring low and two scoring moderate risk, were included with a pooled sample size of *n* = 254 shunted patients, and the pooled random effects size estimate, comparing shunt-responsive to shunt-non-responsive patients, was − 0.10 SMD (CI 95%: − 1.03–0.82), with *t* =  − 0.35 (*p* = 0.75) (Fig. 4). For lumbar CSF P-Tau, four studies were included [4, 33, 51, 74], two scoring low and two moderate risk of bias, with a pooled sample size of *n* = 254 shunted patients, and the pooled random effects size estimate was − 0.55 SMD (CI 95%: − 1.06–(− 0.03)), with *t* =  − 3.40 (*p* = 0.04) (Fig. 5). For T-Tau six studies [4, 33, 51, 70, 73, 74], three scoring low, two moderate, and one scoring critical risk of bias, with a pooled sample size of *n* = 310 shunted patients, were included (one ventricular CSF [70], five lumbar CSF [4, 33, 51, 73, 74], and the pooled random effects size estimate was − 0.50 SMD (CI 95%: − 0.88–(− 0.12)), with *t* =  − 3.34 (*p* = 0.02) (Fig. 6). Overall, the meta-analyses indicated significantly higher levels of CSF P-Tau and T-Tau in shunt-non-responsive than shunt-responsive iNPH subjects (*p* < 0.05), but not for amyloid-β 1–42.

### Sensitivity analysis and meta-regression

A sensitivity analysis was performed by firstly omitting studies with “critical” overall risk of bias on the ROBINS-I tool [69] (Table 1, Table 2, Table 3). The only study that fulfilled this criterion was Tullberg et al. [73], which is one of the studies included in the T-Tau subgroup analysis. Hence, it was omitted in an additional meta-analysis for T-Tau (Supplementary Fig. 1). The meta-analysis yielded a SMD of − 0.56 (95% CI: − 0.98–(− 0.14)), *p* = 0.02. Hence, this study did not affect the overall statistical validity of the initial meta-analysis for T-Tau, as the SMD remained similar to the original SMD (Fig. 6) and the *p*-value remained significant. Subsequently, a single-variate meta-regression was performed for each biomarker (T-Tau, P-Tau, and amyloid-β 1–42). The meta-regressions scored the influence of all covariates on the overall effect size of each biomarker (standard mean difference) to be insignificant for P-Tau and amyloid-β 1–42 (Table 4). However, for T-Tau, the explicit inclusion of patients with neurological comorbidities was found to be significant, with a regression coefficient of − 0.6768 (95% CI: − 1.1243–(− 0.2294)), *p* = 0.0137 (Table 4, for graphical visualisation, see Supplementary Fig. 2). This implies that the inclusion of neurologically comorbid patients negatively skewed the SMD of CSF T-Tau levels between shunt responders and shunt non-responders. To further assess the impact of the covariate “neuro,” multiple multi-variate meta-regression models for T-Tau were built using the covariate “neuro” in combinations with the other covariates (Table 5). For most combinations, doing this rendered the regression coefficient of “neuro” to be non-significant (*p* > 0.05). However, in combination with the covariates “sample” and “date”, the regression coefficient of “neuro” yielded 1.0409 (CI 95%: 0.3674–1.7143), which was significant at *p* = 0.0219. This implies that in this combination, “neuro” positively skewed the SMD of the T-Tau meta-analysis. Finally, to assess the statistical effect of “neuro” on the effect size of T-Tau for shunt response prediction, another subgroup meta-analysis for T-Tau was performed, and now the studies that included neurologically comorbid patients were omitted, namely Ågren-Wilsson et al. [3] and Migliorati et al. [51]. However, this did not have a strong effect, as the SMD remained similar at − 0.36 (CI 96%: − 0.68–(− 0.04)) and remained significant at *p* = 0.04 (Supplementary Fig. 3).

**Table 4:** Mixed-effects single-variate meta-regression

	Total-Tau	Phosphorylated-Tau	Amyloid-β 1–42
~ Covariates	Regression coefficients		
~ *age*	0.0119 (− 0.1361–0.1599)	− 0.0503 (− 0.4015–0.3010)	− 0.0410 (− 0.6861–0.6041)
~ *females*	2.0664 (− 2.4792–6.6121)	9.0232 (− 42.5615–60.6079)	23.9811 (− 26.9236–74.8857)
~ *sample*	0.0119 (− 0.0098–0.0144)	0.0067 (− 0.0055–0.0188)	− 0.0070 (− 0.0335–0.0475)
~ *date*	0.0289 (− 0.0333–0.0910)	− 0.0070 (− 0.1484–0.1343)	− 0.0358 (− 0.2749–0.2033)
~ *srm*	0.0119 (− 0.4259–1.1673)	− 0.1477 (− 2.0210–1.7255)	− 0.4444 (− 3.6846–2.7957)
~ *neuro*	− 0.6768 * [*p* = 0.018] (− 0.0098–0.0144)	0.1477 (− 1.7255–2.0210)	0.4444 (− 2.7957–3.6846)
~ *dropout*	0.1298 (− 1.1618–1.4214)	− 0.3633 (− 2.3627–1.6361)	0.0663 (− 3.5731–3.7057)

The results of the meta-regression of the meta-analyses of Total-Tau, Phosphorylated-Tau, and Amyloid-β 1–42, for each of the covariates (age, females, sample, srm, neuro, dropout) as independent variable to the dependent variable standard mean difference. In round brackets is the 95% confidence intervals. If significance is yielded (denoted with * and bold regression coefficient), the *p*-value of the regression coefficient is shown in squared bracket only if significant, otherwise assume non-significance. Significance is assumed for *p* < 0.05. The covariates age of the patient population (age), the proportion of females in percentage of overall population sample (females), the sample size (sample), the date of publication (date), the method of shunt response measurement (srm), explicit inclusion of patients with neurological comorbidities (neuro), and the dropout rate (dropout) for each study. The different explanatory variables were calculated singularly as sole covariates in separate meta-regressions.

**Table 5:** Mixed-effects multi-variate meta-regression

	Total-Tau
~ Covariates	Regression coefficient of *neuro* only
~ *neuro* + *age* + *females*	0.4641 (− 0.4361 1.3644)
~ *neuro* + *sample* + *date*	1.0409 * [*p* = 0.0219] (0.3674–1.7143)
~ *neuro* + *females* + *date*	0.0119 (− 0.0098–0.0144)
~ *neuro* + *age* + *date*	0.8769 (− 0.3688–2.1226)
~ *neuro* + *females* + *sample*	0.0604 (− 0.2397–1.2943)

The results of the meta-regression of the meta-analyses of Total-Tau, Phosphorylated-Tau, and Amyloid-Beta 1–42, for the covariate “neuro” in combination with the other covariates (age, females, sample, srm, dropout) as independent variable to the dependent variable standard mean difference. In round brackets is the 95% confidence intervals. If significance is yielded (denoted with * and bold regression coefficient), the *p*-value of the regression coefficient is shown in squared bracket if significant only, otherwise assume non-significance. Significance is assumed for *p* < 0.05. The covariates age of the patient population (age), the proportion of females in percentage of overall population sample (females), the sample size (sample), the date of publication (date), the method of shunt response measurement (srm), explicit inclusion of patients with neurological comorbidities (neuro), and the dropout rate (dropout) for each study. The different explanatory variables were calculated by combining three covariates in multi-variate meta-regressions.

### Albatross plot

**Fig. 7:**
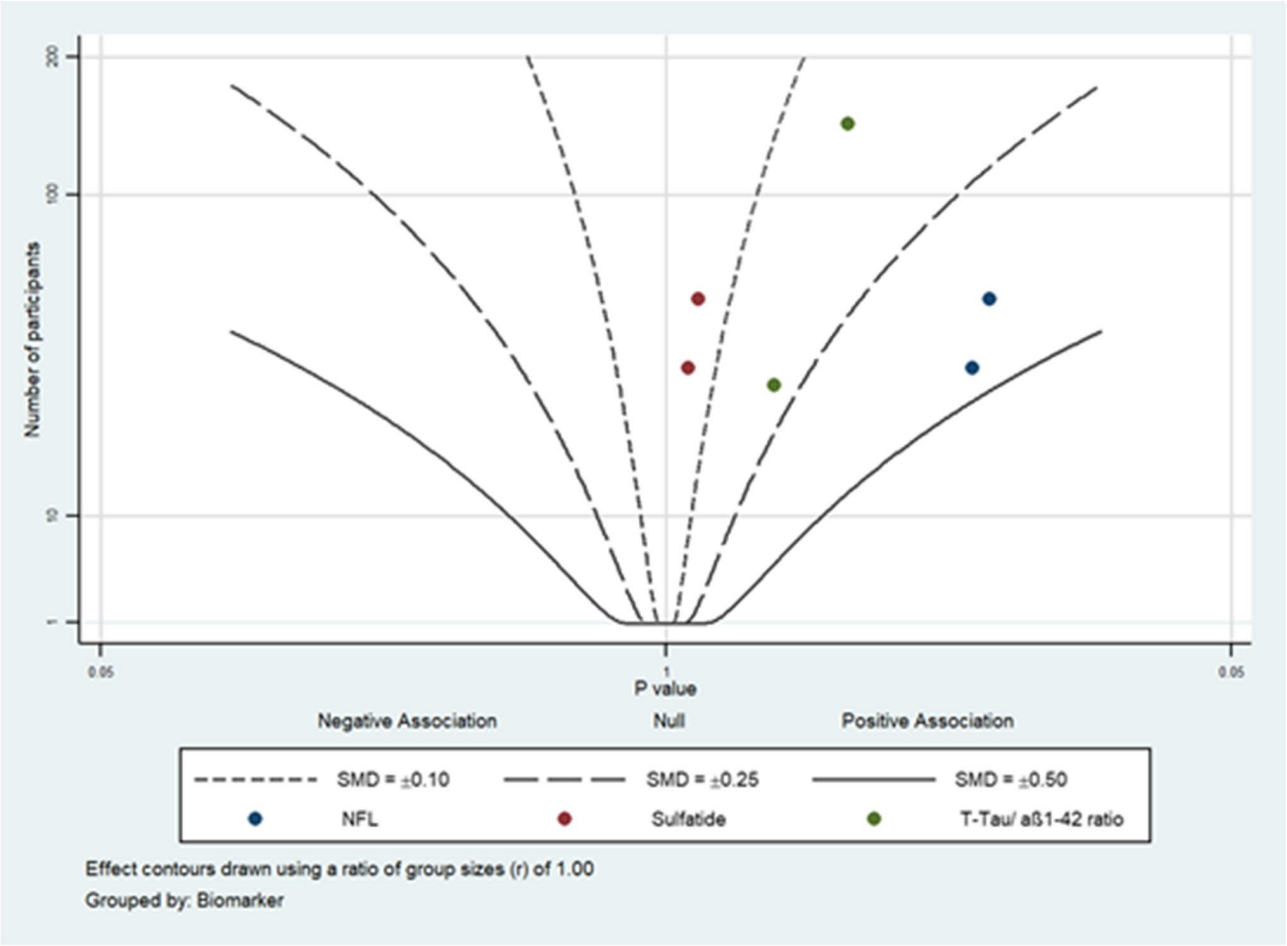
An albatross plot indicating and visualising the effect size as standard mean difference (SMD) of neurofilament light (NFL), sulfatide, and Total-Tau (T-Tau)/amyloid-β 1–42 (aβ 1–42) ratio levels in lumbar CSF samples of shunt responder (S-R) versus shunt non-responder (S-NR) iNPH patients is shown, relative to *p*-value on the x-axis and the sample size on the y-axis (*n* = 4 studies). Three differently drawn lines indicate different SMD levels as outlined in the box. Each biomarker has its own-coloured dot as shown in the box. Each dot represents a single study for the respective biomarker. Studies included for NFL: Ågren-Wilsson et al. (2007) [4], Tullberg et al. (2008) [73]. Studies included for Sulfatide: Ågren-Wilsson et al. (2007) [4], Tullberg et al. (2008) [73]. Studies included for T-Tau/ amyloid-β 1–42: Craven et al. (2017) [15], Hong et al. (2018) [33]. All markers are increased in the S-R group compared to S-NR group, but the difference is not statistically significant

An albatross plot indicating and visualising the effect size as standard mean difference (SMD) was synthesised for studies that met the inclusion criteria for albatross plotting (studies per biomarker *n* = 2), but not for the meta-analysis (studies per biomarker *n* =  > 2). Four studies were included: two with low, one with moderate, and one with critical risk of bias. The biomarkers displayed in the albatross plot were NFL [4, 73], Sulfatide [4, 73], and T-Tau/ amyloid-β 1–42 ratio levels [15, 33] in lumbar CSF samples of shunt-responsive patients compared to shunt-non-responsive patients (Fig. 7). As can be seen on the graph, all markers are increased in shunt-responsive patients compared to shunt-non-responsive; however, all studies are insignificant (*p* > 0.05). Sulfatide is, in numeric terms, the most insignificant biomarker for differentiating between shunt-responsive and shunt-non-responsive patients and has an SMD between 0 and 0.15, followed by T-Tau/ amyloid-β 1–42 ratio with SMD between 0.15 and 0.25, and lastly, and performing best, is NFL with SMD between 0.25 and 0.5.

### Measurement of shunt response

The literature refers to several ways of characterising iNPH patients as shunt responders or shunt non-responders (Tables 1–3). The most common method was the usage of a scoring system before and after the shunt procedure used by nine studies [1, 15, 33, 51, 52, 58, 65, 70, 73]. If the scores had improved by a certain number, then the patient was categorised as shunt-responsive. The most common scale/scoring system used was the iNPH scale, used by Abu Hamdeh et al. (2018) [1], Hong et al. (2018) [33], Migliorati et al. (2021) [51], Minta et al. (2021) [52], Patel et al. (2012) [58], and Vanninen et al. (2021) [74]. Tarnaris et al. (2011) [70] used the Black Grading Scale (BGS) which stratified patients by how much their iNPH grading score improved. Those with scores of “Excellent”, “Good”, and “Fair” were considered shunt responders. Tullberg et al. (2008) [73] utilised a protocol they devised in previous studies that was also a grading scale that incorporated the mini-mental state examination (MMSE), psychometrics, balance, and continence. Craven et al. (2017) [15] used the Wechsler Adult Intelligence Scale as a validated outcome measure [76]. Pyykkö et al. (2012) [60] considered an improvement in any of the core symptoms as positive shunt response—these were improvements in gait, continence, or memory. Ågren-Wilsson et al. (2007) [4] only looked at improvement in gait as means to classify a patient as shunt-responsive.

### Complications

The included studies did not specify complications related to obtaining ventricular or lumbar CSF for biomarker analysis; the reported complications were only related to ventriculoperitoneal shunt insertion. Two patients required a shunt revision due to a misplaced proximal catheter outside the ventricle or distal catheter dislocation as visualised by post-operative computer tomography in the study performed by Abu Hamdeh et al. (2018) [1]. In the same study, two patients experienced catheter over-drainage, which was rectified by increasing the shunt valve pressure setting [1]. In the study by Migliorati et al. (2021) [51], two patients experienced infection at the catheter site and therefore had the shunt removed. In that study, of the total study population, 24 patients were lost to follow-up [51]. Three patients died within 1 year of shunt surgery [33, 51]. Two patients died before follow-up [15].

## Discussion

The main finding of this systematic review and meta-analysis is that there were significantly increased CSF levels of T-Tau and P-Tau in iNPH patients who do not respond to shunt surgery. On the other hand, CSF levels of amyloid-β 1–42 did not differ significantly between shunt non-responders and shunt responders.

The presence of increased levels of the Tau proteins in the CSF is an indicator of neurodegeneration. Tau proteins are abundant in neurons and help maintain axon microtubule skeleton stability. Pathologically elevated levels of particularly P-Tau, a hyperphosphorylated form of Tau, have previously been associated with neurodegenerative disorders such as Alzheimer’s and Parkinson’s diseases [43]. Hence, multiple studies have tried to identify the value of P-Tau and T-Tau in iNPH shunt response prediction, albeit with mostly insignificant differences in levels between shunt responders and shunt non-responders [1, 4, 15, 33, 51, 58, 70, 73, 74]. The authors believe the latter to be due to a sample size error in these studies. Upon pooling all study data on P-Tau and T-Tau, respectively, our meta-analysis found that T-Tau and P-Tau levels were significantly increased in shunt-non-responsive iNPH (*p* = 0.02 and *p* = 0.04, respectively). This discrepancy between Tau protein levels may have several explanations. Elevated levels of Tau protein could be an early manifestation of AD [1], which may have a negative impact on the patient's performance in post-operative neurological assessment tests, weakening or completely masking the associated positive change in symptomology in iNPH pathology after CSF diversion. Human in vivo tracer studies showed impaired clearance of a CSF tracer in iNPH patients [23, 63], which may be one mechanism behind increased CSF levels of metabolites such as Tau. In line with this, Migliorati et al. (2021) [51] hypothesised that higher levels of P-Tau and T-Tau may arise from CSF stasis and subsequent aggregation of toxic Tau protein types in patients with long-standing iNPH or progressed disease. It is argued that in these scenarios, irreversible parenchymal damage is present, which hinders response to shunt surgery. However, none of the existing theories regarding elevated Tau levels in shunt non-responders has been proven, and thus more evidence is needed to consolidate them. In line with the findings of our meta-analysis, Migliorati et al. (2021) [51] went further and examined the diagnostic efficiency of P-Tau and found that the best cut-off for differentiating shunt responders from shunt non-responders was 32.2 ng/l, with a sensitivity of 81.8% and a specificity of 72.4%. Similarly, for T-Tau the best cut-off identified was 233.9 ng/l with a sensitivity and specificity respectively of 81.8% and 72.4%. However, given that this is a single-study finding, as well as the fact that Migliorati et al. (2021) [51] excluded patients comorbid with iNPH mimics from their study which limits the generalisability of their findings, further studies using ROC analyses must be conducted to assess the diagnostic efficiency of Tau biomarkers more reliably. Nonetheless, the results from our meta-analyses regarding Tau levels were also consolidated in brain biopsy studies [1], in which shunt non-responders had higher levels of Tau protein than shunt responders. Given these findings and the fact that the relative complication risk of CSF removal is not increased by biomarker analysis, as iNPH patients invariably undergo some form of CSF removal, the inclusion of biochemical markers in the shunt response prediction pathway of iNPH is logical and inevitable. Overall, the authors advocate for more research on the sensitivity and specificity of specifically the combined use of T-Tau and P-Tau CSF levels, as well as their associated ratios with amyloid-β 1–42, including specific cut-off levels. However, given the current lack of evidence on diagnostic efficiency and cut-offs, the authors do not recommend using CSF Tau protein biomarkers as sole predictors but as complementary variables, using the cut-off proposed by Migliorati et al. (2021) [51], alongside robustly proven clinical predictors such as intracranial pressure monitoring (ICPM) [19–22, 24, 25] and extended lumbar drainage (ELD) [71].

CSF amyloid-β 1–42 has previously been reported to be lower in AD, as a significant proportion of β-amyloid aggregates are fused into plaque fibrils, with particularly amyloid-β 1–42 having an aggregation tendency due to its highly hydrophobic nature [6]. Hence, it was hypothesised whether this biomarker may be lower in shunt non-responsive iNPH [1, 51]. Our meta-analysis found no significant difference in lumbar CSF amyloid-β 1–42 between shunt responders and shunt non-responders (*p* = 0.75). However, the analysis only included three studies; hence, the findings are limited by the low sample size. Migliorati et al. (2021) [51] reported a sensitivity of 72.7% and 79.3% with an optimal cut-off at 731.7 ng/l for CSF amyloid-β 1–42 when used to predict shunt response, but further studies are needed to consolidate their findings. Overall, the authors do not recommend CSF amyloid-β 1–42 to be used as a variable in shunt response prediction, until more research proves a significant difference between shunt responders and shunt non-responders.

The existing literature on all other CSF biomarkers (ECM, VIP, LRG, NFL, Sulfatide, Albumin, Sulfatide) is extremely sparse, with none of these having been examined by more than two of the included studies; hence, it is not possible to make a robust conclusion on their use in the prediction of shunt-responsive iNPH. However, the rationale of using these biomarkers is often similar to the use of the Tau proteins and amyloid-β 1–42, with levels of nearly all the miscellaneous biomarkers, particularly sulfatide [11], being reported to be significantly altered in AD patients; hence, future research is highly warranted to examine their use further. The use of proteomics techniques, such as two-dimensional electrophoresis coupled with MALDI TOF MS technique for the analysis of protein biomarkers, did not yield any statistically significant differences between shunt responders and shunt non-responders, and neither did the genotyping of blood samples. However, as the mentioned proteomics technique is the current gold standard in terms of accuracy to analyse proteins, the authors recommend future research to use this technique when analysing CSF biomarkers for shunt-responsive iNPH. Similarly, the use of genetic analysis in this context must be elucidated further [38], as most iNPH mimics have a proven genetic etiological basis, particularly Alzheimer’s [9] and Parkinson’s [40] diseases. However, the ethical implications of genetic testing are complex and must be managed carefully.

An important weakness of the existing literature on biomarkers for iNPH shunt response prediction is the lack of investigator blinding. In fact, only two studies [1, 15] reported blinding, with the remaining 11 studies reporting no blinding at all. One of these studies [58] only used a single investigator for data collection, which incurs a critical source of bias. Furthermore, two of the included studies had quite a significant dropout [33, 58], with Patel et al. (2012) [58] having an approximately 25% dropout of the initial cohort, rates which quite possibly incur a significant source of selection bias. In these studies, patients who did not respond to CSF removal via lumbar infusion test or CSF tap test likely dropped out and consequently leading to a skewed sample size in the shunt non-responder group. The negative effect of this on this meta-analysis is quite apparent in the analysis T-Tau (Fig. 6), with 109 patients in the shunt non-responder group, compared to 201 patients in the shunt responder group. The unequal sample size may have affected the statistical power of the analysis. Moreover, three studies [1, 51, 60] excluded patients with neurodegenerative comorbidities categorically from the studies, which represent a grave methodological flaw that undermines the generalisability and clinical usefulness of their study results, as iNPH is a neurodegenerative disease itself with close overlap with Alzheimer’s [46] and Parkinson’s [54] diseases. Other studies [33, 65, 74] did not exclude iNPH patients with neurodegenerative conditions; however, they did not explicitly mention them at all when outlining patient characteristics even though neurological comorbidities are a significant confounder. This was reflected in the univariate meta-regression for T-Tau (Table 4), which showed that studies that included patients with neurological comorbidities skewed the SMD of T-Tau levels between shunt responders and shunt non-responders negatively. Even though it did not affect the overall findings of the T-Tau subgroup meta-analysis, the regression coefficient was significant (Table 4). Hence, in the future, studies should include patients with neurological comorbidities for more generalisable findings—however, the authors recommend that in the statistical analysis, patients with neurological comorbidities should be separately analysed and reported to allow for a fair comparison. Tullberg et al. (2008) [73] pooled shunt response results of iNPH patients and secondary iNPH in the final step of statistical analysis, constituting a critical error, as it makes it extremely questionable whether the study results apply to either pathology cohort, which are both completely different from each other in terms of disease aetiology. This may explain why their study, the only study scoring overall critical risk of bias (Table 3), was treated as an outlier in the meta-analysis and hence not included in the overall *t* statistic (Fig. 6). Finally, none of the studies, except Craven et al. (2007) [15] and Migliorati et al. (2021) [51], provided calculations on diagnostic efficiency (area under curve value, diagnostic odds ratio, sensitivity, specificity) of the biomarkers at a certain cut-off level, which makes it impossible to perform a meta-analysis on the diagnostic utility of the biomarkers to predict shunt response.

### Limitations

The key limitation of this study, because of the methodological weakness of the included literature, is that our meta-analysis is a pooled effect size (SMD) analysis. While this type of analysis can highlight significant differences between shunt responders and shunt non-responders, it cannot provide information on which cut-off to choose and what the diagnostic accuracy (overall sensitivity and specificity) of each biomarker is. Future research must provide data on the true negatives, false negatives, true positives, and false positives transparently, in order to run a robust meta-analysis of diagnostic accuracy [71]. Overall, the number of studies per biomarkers was relatively low, particularly for amyloid-β 1–42 (*n* = 3), which limits the validity of the meta-analysis. More robust studies are needed in this field in the future to allow for more reliable pooling of results for all biomarkers included in our meta-analysis and those that were excluded from it.

## Conclusion

The lumbar CSF levels of P-Tau and T-Tau were significantly increased in shunt-non-responsive iNPH. Other CSF or venous biomarkers, including amyloid-β 1–42, did not differentiate shunt-responsive from shunt non-responsive iNPH. More studies on Tau proteins, which not only examine differences in total levels but also sensitivity and specificity at specific cut-off levels, are needed. This would allow for a robust analysis of diagnostic efficiency and clearer guidance on the use of CSF Tau proteins for predicting shunt response in iNPH, including the best cut-off values. Similarly, further research, employing uniform shunt response criteria, must continue to examine the other CSF proteins (NFL, Albumin, VIP, Sulfatide, LRG, ECM proteins, Clusterin), as well as genotyping and proteomics analysis, to establish an adequate sample size for a meta-analysis.

## Supplementary Information

Below is the link to the electronic supplementary material.

Supplementary file1 (PDF 604 KB)

## Abbreviations

5-HIAA: 5-Hydroxy-indoleacetic acid
a1b-GP: Alpha 1 beta glycoprotein
a2-HS-GP: Alpha 2 Heremans–Schmid glycoprotein
AD: Alzheimer’s disease
aβO10-20: Amyloid-β oligomers, consisting of 10–20 monomers
Amyloid-β 1-42: Amyloid beta 1.42
Apo E: Apolipoprotein E
Apo J: Apolipoprotein J
AUROC: Area under the receiving operating characteristic
BGS: Black Grading Scale
CSF: Cerebrospinal fluid
ELD: Extended lumbar drainage
FU: Follow-up
GABA: Gamma amino butyric acid
GFAP: Glial fibrillary acid protein
HMPG: 4-Hydroxy-3-Methoxyphenylglycol
HVA: Homovanillic acid
ICPM: Intracranial pressure monitoring
iNPH: Idiopathic normal pressure hydrocephalus
LP: Lumbar puncture
LRG: Leucine-rich alpha-2 glycoprotein
LTFU: Lost-to-follow-up
MALDI TOF MS: 2D-Gel electrophoresis and matrix-assisted laser desorption/ionisation and time of flight mass spectrometry desorption/ionisation and time of flight mass spectrometry
MMP: Matrix metalloproteinases
MMSE: Mini-mental state examination
MRS: Modified Rankin Scale
NFL: Neurofilament triplet protein
NPY: Neuropeptide Y
OCEBM: Oxford Centre of Evidence Based Medicine Levels of Evidence
PRISMA: Preferred Reporting Items for Systematic Reviews and Meta-Analyses
P-Tau: Phosphorylated Tau protein
ROBINS-I: Risk Of Bias in Non-randomised Studies of Interventions
ROC: Receiver operating characteristic (ROC)
SMD: Standardised mean difference
S-R: Shunt responder
S-NR: Shunt non-responder
TIMP-1: Tissue inhibitor matrix metalloproteinase 1
TT: Tap test
T-Tau: Total Tau protein
VIP: Vasoactive intestinal peptide
