# Supplementary material for: Cerebrospinal fluid and venous biomarkers of shunt-responsive idiopathic normal pressure hydrocephalus: a systematic review and meta-analysis
Source: Acta Neurochir (Wien). 2022 Mar 1;164(7):1719–46. doi: 10.1007/s00701-022-05154-5 (PMC9233649; doi:10.1007/s00701-022-05154-5)
Supplement: Supplementary file 1 — Supplementary file1 (PDF 604 KB) [file 701_2022_5154_MOESM1_ESM.pdf]

Supplementary material

Cerebrospinal fluid and venous biomarkers of shunt-responsive idiopathic normal pressure hydrocephalus: a systematic review and meta-analysis

Santhosh G. Thavarajasingam[1]; Mahmoud El-Khatib[1]; Kalyan V. Vemulapalli[1]; Hector A. Sinzinkayo Iradukunda[1]; Joshua Laleye[1], Salvatore Russo[2]; Christian Eichhorn[3]; Per K. Eide[4,5]

1. Faculty of Medicine, Imperial College London, London, United Kingdom.
2. Department of Neurosurgery, Imperial College Healthcare NHS Trust, London, United Kingdom
3. Division of Internal Medicine – University Hospital Basel, Basel, Switzerland
4. Department of Neurosurgery, Oslo University Hospital – Rikshospitalet, Oslo, Norway
5. Institute of Clinical Medicine, Faculty of Medicine, University of Oslo, Oslo, Norway

Contents

Supplementary Figure 1: 3
Supplementary Figure 2: 4
Supplementary Figure 3: 5
Supplementary Table 1: 6
Supplementary Table 2: 9
Supplementary Table 3: 10
Supplementary Table 4: 25
Supplementary Table 5: 25
Supplementary Table 6: 25
References 26

Study	S-R Total	S-R Mean	S-R SD	S-NR Total	S-NR Mean	S-NR SD	SMD	95%-CI	Weight
Tarnaris et al.	17	550.97	551.6500	4	1086.00	347.1900	-1.02	[-2.16; 0.12]	7.5%
Ågren-Wilsson et al.	33	164.00	115.0000	22	269.00	93.0000	-0.98	[-1.55; -0.41]	22.9%
Migliorati et al.	15	161.10	185.6000	35	245.00	137.9750	-0.55	[-1.16; 0.07]	20.6%
Hong et al.	17	141.90	40.8000	14	177.20	126.7000	-0.39	[-1.11; 0.32]	16.5%
Vanninen et al.	91	190.40	86.8000	27	211.30	75.9000	-0.25	[-0.68; 0.18]	32.5%
**Random effects model**	**173**			**102**			**-0.56**	**[-0.98; -0.14]**	**100.0%**
**Prediction interval**								**[-1.35; 0.23]**	

Heterogeneity: $I^2 = 19\%$, $p = 0.29$

Overall statistical result of model: $t_4 = -3.66$ ($p = 0.02$)

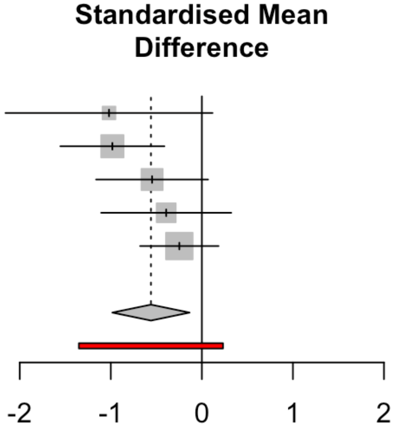

Supplementary Figure 1: In *Fig. A*, as part of the sensitivity analysis of the meta-analysis, a forest plot indicating and visualizing the effect size in standard mean difference (SMD) of Total-Tau (T-Tau) levels in lumbar (n=4) and ventricular (n=1, Tarnaris et al. (2011) [6]) samples of shunt responder (S-R) versus shunt non-responder (S-NR) iNPH patients is shown (n = 5 studies). This meta-analysis for T-Tau does not include the study by Tullberg et al. (2008) [7], which is the only study on T-Tau which scored “critical” level of risk of bias on the ROBINS-I tool [5] (Table 2).The size of the grey square of the SMD visual correlates to study sample size and the straight line indicated the confidence interval. The diamond at the bottom indicates the overall pooled effect. The red bar below it indicates the prediction interval. Heterogeneity is indicated by the chi-squared statistic (I^2) with associated p-value. The 95% confidence intervals (CI) are shown in squared bracket ([ ]). Furthermore, for every study the following are displayed: author, total number of S-R and their respective mean level and standard deviation (SD) of T-Tau lumbar CSF levels, as well as the respective values for S-NR, weighting of each study in percentage.

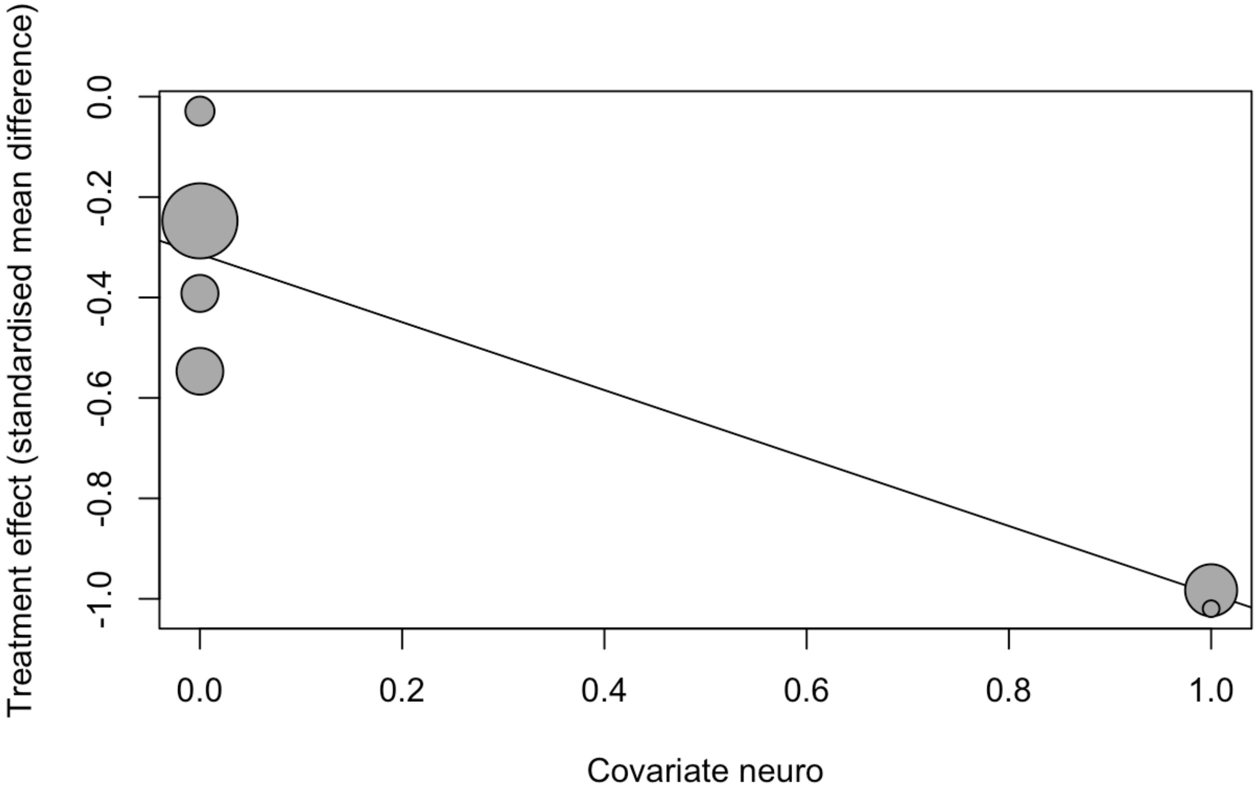

Supplementary Figure 2: In *Fig. B*, a bubble plot is shown which visualises the result of the meta-regression on the meta-analysis of T-Tau (n= 6 studies) of the treatment effect (standard mean difference) relative to the covariate "neuro". This covariate implies the explicit inclusion of patients with neurological co-morbidities. The size of the bubbles correlates to the sample size. The two studies on the bottom right are Ågren-Wilsson et al. (2007) [1] and Migliorati et al. (2021) [3], which are the only two studies included in this subgroup analysis that explicitly included patients with neurological co-morbidities. The others, on the top left, are Hong et al. (2018) [2], Tarnaris et al. (2011) [6], Tullberg et al. (2008) [7] and Vanninen et al. (2021) [8]

Study	S-R Total	S-R Mean	S-R SD	S-NR Total	S-NR Mean	S-NR SD	SMD	95%-CI	Weight
Tarnaris et al.	17	550.97	551.6500	4	1086.00	347.1900	-1.02	[-2.16; 0.12]	6.3%
Migliorati et al.	15	161.10	185.6000	35	245.00	137.9750	-0.55	[-1.16; 0.07]	21.6%
Hong et al.	17	141.90	40.8000	14	177.20	126.7000	-0.39	[-1.11; 0.32]	16.0%
Vanninen et al.	91	190.40	86.8000	27	211.30	75.9000	-0.25	[-0.68; 0.18]	44.1%
Tullberg et al.	28	267.00	296.0000	7	275.00	165.0000	-0.03	[-0.86; 0.80]	11.9%
**Random effects model**	**168**			**87**			**-0.36**	**[-0.68; -0.04]**	**100.0%**
**Prediction interval**								**[-0.73; 0.01]**	

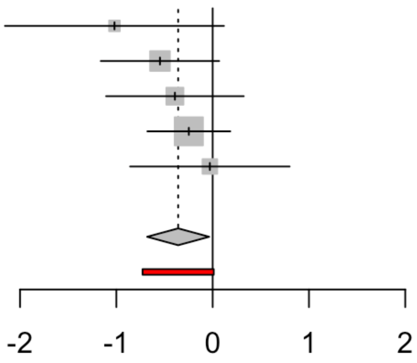

Heterogeneity: $I^2 = 0\%$, $p = 0.64$

Overall statistical result of model: $t_4 = -3.09$ ($p = 0.04$)

Supplementary Figure 3: In *Fig. C*, as part of the sensitivity analysis of the meta-analysis, a forest plot indicating and visualizing the effect size in standard mean difference (SMD) of Total-Tau (T-Tau) levels in lumbar (n=5) and ventricular (n=1, Tarnaris et al. (2011) [6]) samples of shunt responder (S-R) versus shunt non-responder (S-NR) iNPH patients is shown (n = 5 studies). This meta-analysis for T-Tau does not include Ågren-Wilsson et al. (2007) [1] and Migliorati et al. (2021) [3] , which are the only two studies included in this subgroup analysis that explicitly included patients with neurological co-morbidities and caused a significant result in the meta-regression of the Total-Tau meta-analysis [5] (Figure B, Supplementary Material and Table 4, main article).The size of the grey square of the SMD visual correlates to study sample size and the straight line indicated the confidence interval. The diamond at the bottom indicates the overall pooled effect. The red bar below it indicates the prediction interval. Heterogeneity is indicated by the chi-squared statistic (I^2) with associated p-value. The 95% confidence intervals (CI) are shown in squared bracket ([ ]). Furthermore, for every study the following are displayed: author, total number of S-R and their respective mean level and standard deviation (SD) of T-Tau lumbar CSF levels, as well as the respective values for S-NR, weighting of each study in percentage.

Supplementary Table 1: The Preferred Reporting Items for Systematic Reviews and Meta-Analyses (PRISMA) 2020 statement comprises a 27-item checklist addressing the introduction, methods, results and discussion sections of this systematic review report [4].

Section and Topic	Item #	Checklist item	Location where item is reported
**TITLE**			
Title	1	Identify the report as a systematic review.	Page 1.
**ABSTRACT**			
Abstract	2	See the PRISMA 2020 for Abstracts checklist.	Page 2.
**INTRODUCTION**			
Rationale	3	Describe the rationale for the review in the context of existing knowledge.	Page 3-4.
Objectives	4	Provide an explicit statement of the objective(s) or question(s) the review addresses.	Page 4.
**METHODS**			
Eligibility criteria	5	Specify the inclusion and exclusion criteria for the review and how studies were grouped for the syntheses.	Page 5.
Information sources	6	Specify all databases, registers, websites, organisations, reference lists and other sources searched or consulted to identify studies. Specify the date when each source was last searched or consulted.	Page 5.
Search strategy	7	Present the full search strategies for all databases, registers and websites, including any filters and limits used.	Supplementary Material: Table B; Page 4.
Selection process	8	Specify the methods used to decide whether a study met the inclusion criteria of the review, including how many reviewers screened each record and each report retrieved, whether they worked independently, and if applicable, details of automation tools used in the process.	Page 5-6.
Data collection process	9	Specify the methods used to collect data from reports, including how many reviewers collected data from each report, whether they worked independently, any processes for obtaining or confirming data from study investigators, and if applicable, details of automation tools used in the process.	Page 6.
Data items	10a	List and define all outcomes for which data were sought. Specify whether all results that were compatible with each outcome domain in each study were sought (e.g. for all measures, time points, analyses), and if not, the methods used to decide which results to collect.	Page 6.
	10b	List and define all other variables for which data were sought (e.g. participant and intervention characteristics, funding sources). Describe any assumptions made about any missing or unclear information.	Page 6.
Study risk of bias assessment	11	Specify the methods used to assess risk of bias in the included studies, including details of the tool(s) used, how many reviewers assessed each study and whether they worked independently, and if applicable, details of automation tools used in the process.	Page 6.
Effect measures	12	Specify for each outcome the effect measure(s) (e.g. risk ratio, mean difference) used in the synthesis or presentation of results.	Page 6.
Synthesis methods	13a	Describe the processes used to decide which studies were eligible for each synthesis (e.g. tabulating the study intervention characteristics and comparing against the planned groups for each synthesis (item #5)).	Page 6.
	13b	Describe any methods required to prepare the data for presentation or synthesis, such as handling of	Page 6.

Section and Topic	Item #	Checklist item	Location where item is reported
		missing summary statistics, or data conversions.	
	13c	Describe any methods used to tabulate or visually display results of individual studies and syntheses.	Page 6.
	13d	Describe any methods used to synthesize results and provide a rationale for the choice(s). If meta-analysis was performed, describe the model(s), method(s) to identify the presence and extent of statistical heterogeneity, and software package(s) used.	Page 6-7.
	13e	Describe any methods used to explore possible causes of heterogeneity among study results (e.g. subgroup analysis, meta-regression).	Page 7-8.
	13f	Describe any sensitivity analyses conducted to assess robustness of the synthesized results.	Page 7-8.
Reporting bias assessment	14	Describe any methods used to assess risk of bias due to missing results in a synthesis (arising from reporting biases).	Page 6
Certainty assessment	15	Describe any methods used to assess certainty (or confidence) in the body of evidence for an outcome.	Page 6.
**RESULTS**			
Study selection	16a	Describe the results of the search and selection process, from the number of records identified in the search to the number of studies included in the review, ideally using a flow diagram.	Figure 1; page 35
	16b	Cite studies that might appear to meet the inclusion criteria, but which were excluded, and explain why they were excluded.	N/A
Study characteristics	17	Cite each included study and present its characteristics.	Tables 1-3; Page 22-32.
Risk of bias in studies	18	Present assessments of risk of bias for each included study.	Tables 1-3; Page 22-32. Figure 2; Page 36.
Results of individual studies	19	For all outcomes, present, for each study: (a) summary statistics for each group (where appropriate) and (b) an effect estimate and its precision (e.g. confidence/credible interval), ideally using structured tables or plots.	Tables 1-3; Page 22-32.<br>Figure 4; page 38. Figure 5, page 39. Figure 6; page 40. Figure 7; page 41.
Results of syntheses	20a	For each synthesis, briefly summarise the characteristics and risk of bias among contributing studies.	Page 13-14.
	20b	Present results of all statistical syntheses conducted. If meta-analysis was done, present for each the summary estimate and its precision (e.g. confidence/credible interval) and measures of statistical heterogeneity. If comparing groups, describe the direction of the effect.	Page 12-14. Figure 4; page 38. Figure 5, page 39. Figure 6; page 40. Figure 7; page 41.
	20c	Present results of all investigations of possible causes of heterogeneity among study results.	Figure 3; page 37.
	20d	Present results of all sensitivity analyses conducted to assess the robustness of the synthesized results.	Pages 13-14. Tables 4-5; pages 33-34.

Section and Topic	Item #	Checklist item	Location where item is reported
Reporting biases	21	Present assessments of risk of bias due to missing results (arising from reporting biases) for each synthesis assessed.	Risk of bias (ROBINS-1) Column, section E. Tables 1-3; Page 22-32.
Certainty of evidence	22	Present assessments of certainty (or confidence) in the body of evidence for each outcome assessed.	Level of evidence (OECBM) column. Tables 1-3; Page 22-32.
**DISCUSSION**			
Discussion	23a	Provide a general interpretation of the results in the context of other evidence.	Pages 16-20.
	23b	Discuss any limitations of the evidence included in the review.	Page 19-20.
	23c	Discuss any limitations of the review processes used.	Page 20.
	23d	Discuss implications of the results for practice, policy, and future research.	Page 17,18-19,20.
**OTHER INFORMATION**			
Registration and protocol	24a	Provide registration information for the review, including register name and registration number, or state that the review was not registered.	Page 5.
	24b	Indicate where the review protocol can be accessed, or state that a protocol was not prepared.	Page 5.
	24c	Describe and explain any amendments to information provided at registration or in the protocol.	N/A
Support	25	Describe sources of financial or non-financial support for the review, and the role of the funders or sponsors in the review.	Page 1.
Competing interests	26	Declare any competing interests of review authors.	Page 1.
Availability of data, code and other materials	27	Report which of the following are publicly available and where they can be found: template data collection forms; data extracted from included studies; data used for all analyses; analytic code; any other materials used in the review.	Supplementary material; Appendix: A-D.

Supplementary Table 2: Search strategy performed on 1st November 2021 including the name of the database, search term(s)/search string used, as well as the publication dates chosen as limiting factors, and the results.

Database	Search terms	Publications dates	Results (n)
MEDLINE	normal pressure hydrocephalus.mp. [mp=ti, ab, hw, tn, ot, dm, mf, dv, kf, fx, dq, nm, ox, px, rx, an, ui, sy]<br>limit 1 to yr="1965 - 2021"	1965-2021	n = 4133
Embase	normal pressure hydrocephalus.mp. [mp=ti, ab, hw, tn, ot, dm, mf, dv, kf, fx, dq, nm, ox, px, rx, an, ui, sy]<br>limit 1 to yr="1965 - 2021"	1965-2021	n = 2891
Scopus	normal AND pressure AND hydrocephalus AND response OR responsiveness OR responder OR predict AND (EXCLUDE (PUBYEAR , 2021) OR EXCLUDE (PUBYEAR , 2020) OR EXCLUDE (PUBYEAR , 2019) OR EXCLUDE (PUBYEAR , 2018) OR EXCLUDE (PUBYEAR , 2022))	1970-2021	n = 4,456
PubMed	(normal pressure hydrocephalus) AND ((response) OR (responsiveness) OR (responder) OR (predict))	1971-2021	n = 1,024
Google Scholar	idiopathic normal pressure hydrocephalus shunt response predict marker	1965-2021	n = 5,850
JSTOR	Normal pressure hydrocephalus	1965-2021	n = 921

Supplementary Table 3: The R code utilised to program the meta-analysis and associated figures, ROB graphs, as well as the meta-regression.

```
#Install relevant packages
install.packages(c("robumeta", "metafor", "dplyr"))
install.packages("meta")
install.packages("dmetar")
install.packages("readxl")
#into library load
library("robumeta")
library("metafor")
library("dplyr")
library("mada")
library("meta")
library("dmetar")
library("readxl")
```

```
# Load TTAU dataset from dmetar (or download and open manually)
library(readxl)
iNPH_Biochem_TTau_Excel <- read_excel("~/Desktop/iNPH Biochem - TTau.xlsx")
View(iNPH_Biochem_TTau_Excel)

# Use metcont to pool results.
meta_ttau <- metacont(n.e = iNPH_Biochem_TTau_Excel$n.SR,
                      mean.e = iNPH_Biochem_TTau_Excel$mean.SR,
                      sd.e = iNPH_Biochem_TTau_Excel$sd.SR,
                      n.c = iNPH_Biochem_TTau_Excel$n.SNR,
                      mean.c = iNPH_Biochem_TTau_Excel$mean.SNR,
                      sd.c = iNPH_Biochem_TTau_Excel$sd.SNR,
                      studlab = iNPH_Biochem_TTau_Excel$Author,
                      data = iNPH_Biochem_TTau_Excel,
                      sm = "SMD",
                      method.smd = "Cohen",
```

```
                      fixed = FALSE,
                      random = TRUE,
                      method.tau = "REML",
                      hakn = TRUE,
                      title = "T-Tau levels in SR vs N-SR")

#numerical visualisation of meta_ttau (note that SR are the experimental group so reference group, so
minus result means it is less in them)

print(meta_ttau)

#make the plot

forest.meta(meta_ttau,
            sortvar = TE,
            predict = TRUE,
            print.tau2 = FALSE,
            leftlabs = c("Author", "g", "SE"),
            lab.e = "S-R",
            lab.c = "S-NR",
            JAMA.pval = FALSE,
            test.overall.random = TRUE,
            label.test.overall.random = "Overall statistical result of model: ")


#meta-regression metareg: single ones (all)

metareg_ttau_females <- metareg(meta_ttau,
        ~ females)
print(metareg_ttau_females)


metareg_ttau_age <- metareg(meta_ttau,
                                 ~ age)
print(metareg_ttau_age)


metareg_ttau_sample <- metareg(meta_ttau,
                             ~ sample)
```

```
print(metareg_ttau_sample)


metareg_ttau_neuro <- metareg(meta_ttau,
                               ~ neuro)
print(metareg_ttau_neuro)


metareg_ttau_dropout<- metareg(meta_ttau,
                              ~ dropout)
print(metareg_ttau_dropout)


metareg_ttau_srm<- metareg(meta_ttau,
                               ~ srm)
print(metareg_ttau_srm)

metareg_ttau_date <- metareg(meta_ttau,
                           ~ date)
print(metareg_ttau_date)
# meta regression step 2: neuro plus others

metareg_ttau_neuroagefemales <- metareg(meta_ttau,
                              ~ neuro + age + females)
print(metareg_ttau_neuroagefemales)

metareg_ttau_neurosampledate <- metareg(meta_ttau,
                                        ~  neuro + sample + date)
print(metareg_ttau_neurosampledate)

metareg_ttau_neurofemalesdate <- metareg(meta_ttau,
                                        ~  neuro + females + date)
print(metareg_ttau_neuroagefemales)

metareg_ttau_neuroagedate <- metareg(meta_ttau,
                                         ~  neuro + age + date)
print(metareg_ttau_neuroagedate)

metareg_ttau_neurosamplefemales <- metareg(meta_ttau,
```

```
                                  ~  neuro + sample + females)

print(metareg_ttau_neurosamplefemales)

#other models were tried, no significance

#plot the metaregression neuro

bubble(metareg_ttau_neuro, studlab = TRUE, scale = TRUE)

#meta-analysis without the study positive for "neuro" (explicitly included neuro patients)

iNPH_Biochem_TTau_Excel2 <- read_excel("~/Desktop/iNPH Biochem - TTau2.xlsx")
View(iNPH_Biochem_TTau_Excel2)

# Use metcont to pool results.
meta_ttau2 <- metacont(n.e = iNPH_Biochem_TTau_Excel2$n.SR,
                      mean.e = iNPH_Biochem_TTau_Excel2$mean.SR,
                      sd.e = iNPH_Biochem_TTau_Excel2$sd.SR,
                      n.c = iNPH_Biochem_TTau_Excel2$n.SNR,
                      mean.c = iNPH_Biochem_TTau_Excel2$mean.SNR,
                      sd.c = iNPH_Biochem_TTau_Excel2$sd.SNR,
                      studlab = iNPH_Biochem_TTau_Excel2$Author,
                      data = iNPH_Biochem_TTau_Excel2,
                      sm = "SMD",
                      method.smd = "Cohen",
                      fixed = FALSE,
                      random = TRUE,
                      method.tau = "REML",
                      hakn = TRUE,
                      title = "T-Tau levels in SR vs N-SR")

#numerical visualisation of meta_ttau (note that SR are the experimental group so reference group, so
minus result means it is less in them)

print(meta_ttau2)

#make the plot
```

```
forest.meta(meta_ttau2,
            sortvar = TE,
            predict = TRUE,
            print.tau2 = FALSE,
            leftlabs = c("Author", "g", "SE"),
            lab.e = "S-R",
            lab.c = "S-NR",
            JAMA.pval = FALSE,
            test.overall.random = TRUE,
            label.test.overall.random = "Overall statistical result of model: ")


#########################################################################################################
##############



# Load P-TAU dataset from dmetar (or download and open manually)

library(readxl)
iNPH_Biochem_PTau_Excel <- read_excel("~/Desktop/iNPH Biochem - PTau.xlsx")
View(iNPH_Biochem_PTau_Excel)

# Use metcont to pool results.
meta_ptau <- metacont(n.e = iNPH_Biochem_PTau_Excel$n.SR,
                      mean.e = iNPH_Biochem_PTau_Excel$mean.SR,
                      sd.e = iNPH_Biochem_PTau_Excel$sd.SR,
                      n.c = iNPH_Biochem_PTau_Excel$n.SNR,
                      mean.c = iNPH_Biochem_PTau_Excel$mean.SNR,
                      sd.c = iNPH_Biochem_PTau_Excel$sd.SNR,
                      studlab = iNPH_Biochem_PTau_Excel$Author,
                      data = iNPH_Biochem_PTau_Excel,
                      sm = "SMD",
                      method.smd = "Cohen",
                      fixed = FALSE,
                      random = TRUE,
                      method.tau = "REML",
                      hakn = TRUE,
                      title = "P-Tau levels in SR vs N-SR")
```

```
#numerical visualisation of meta_ttau (note that SR are the experimental group so reference group, so minus result means it is less in them)

print(meta_ptau)

#make the plot

forest.meta(meta_ptau,
            sortvar = TE,
            predict = TRUE,
            print.tau2 = FALSE,
            leftlabs = c("Author", "g", "SE"),
            lab.e = "S-R",
            lab.c = "S-NR",
            JAMA.pval = FALSE,
            test.overall.random = TRUE,
            label.test.overall.random = "Overall statistical result of model: ")



#meta-regression metareg: single ones (all)

metareg_ptau_females <- metareg(meta_ptau,
                                ~ females)
print(metareg_ptau_females)


metareg_ptau_age <- metareg(meta_ptau,
                            ~ age)
print(metareg_ptau_age)


metareg_ptau_sample <- metareg(meta_ptau,
                               ~ sample)
print(metareg_ptau_sample)


metareg_ptau_neuro <- metareg(meta_ptau,
```

```
                              ~ neuro)
print(metareg_ptau_neuro)


metareg_ptau_dropout<- metareg(meta_ptau,
                               ~ dropout)
print(metareg_ptau_dropout)


metareg_ptau_srm<- metareg(meta_ptau,
                           ~ srm)
print(metareg_ptau_srm)

metareg_ptau_date <- metareg(meta_ptau,
                             ~ date)
print(metareg_ptau_date)


#########################################################################################################
##############



# Load Abeta dataset from dmetar (or download and open manually)

library(readxl)
iNPH_Biochem_Abeta_Excel <- read_excel("~/Desktop/iNPH Biochem - Abeta.xlsx")
View(iNPH_Biochem_Abeta_Excel)

# Use metcont to pool results.
meta_Abeta <- metacont(n.e = iNPH_Biochem_Abeta_Excel$n.SR,
                       mean.e = iNPH_Biochem_Abeta_Excel$mean.SR,
                       sd.e = iNPH_Biochem_Abeta_Excel$sd.SR,
                       n.c = iNPH_Biochem_Abeta_Excel$n.SNR,
                       mean.c = iNPH_Biochem_Abeta_Excel$mean.SNR,
                       sd.c = iNPH_Biochem_Abeta_Excel$sd.SNR,
                       studlab = iNPH_Biochem_Abeta_Excel$Author,
                       data = iNPH_Biochem_Abeta_Excel,
                       sm = "SMD",
```

```
                        method.smd = "Cohen",
                        fixed = FALSE,
                        random = TRUE,
                        method.tau = "REML",
                        hakn = TRUE,
                        title = "Abeta levels in SR vs N-SR")

#numerical visualisation of meta_ttau (note that SR are the experimental group so reference group, so
minus result means it is less in them)

print(meta_Abeta)

#make the plot

forest.meta(meta_Abeta,
            sortvar = TE,
            predict = TRUE,
            print.tau2 = FALSE,
            leftlabs = c("Author", "g", "SE"),
            lab.e = "S-R",
            lab.c = "S-NR",
            JAMA.pval = FALSE,
            test.overall.random = TRUE,
            label.test.overall.random = "Overall statistical result of model: ")


#meta-regression metareg: single ones (all)

metareg_Abeta_females <- metareg(meta_Abeta,
                                 ~ females)
print(metareg_Abeta_females)


metareg_Abeta_age <- metareg(meta_Abeta,
                             ~ age)
print(metareg_Abeta_age)


metareg_Abeta_sample <- metareg(meta_Abeta,
```

```
                                ~ sample)
print(metareg_Abeta_sample)


metareg_Abeta_neuro <- metareg(meta_Abeta,
                               ~ neuro)
print(metareg_Abeta_neuro)


metareg_Abeta_dropout<- metareg(meta_Abeta,
                                ~ dropout)
print(metareg_Abeta_dropout)


metareg_Abeta_srm<- metareg(meta_Abeta,
                            ~ srm)
print(metareg_Abeta_srm)

metareg_Abeta_date <- metareg(meta_Abeta,
                              ~ date)
print(metareg_Abeta_date)

# meta regression step 2: neuro plus others

metareg_ttau_neuroagefemales <- metareg(meta_ttau,
                                        ~ neuro + age + females)
print(metareg_ttau_neuroagefemales)

metareg_ttau_neurosampledate <- metareg(meta_ttau,
                                        ~  neuro + sample + date)
print(metareg_ttau_neurosampledate)

#other models were tried, no significance

#plot the metaregression neuro

bubble(metareg_ttau_neuro, studlab = TRUE, scale = TRUE)

#meta-analysis without the study positive for "neuro" (explicitly included neuro patients)
```

```
iNPH_Biochem_TTau_Excel2 <- read_excel("~/Desktop/iNPH Biochem - TTau2.xlsx")
View(iNPH_Biochem_TTau_Excel2)

# Use metcont to pool results.
meta_ttau2 <- metacont(n.e = iNPH_Biochem_TTau_Excel2$n.SR,
                       mean.e = iNPH_Biochem_TTau_Excel2$mean.SR,
                       sd.e = iNPH_Biochem_TTau_Excel2$sd.SR,
                       n.c = iNPH_Biochem_TTau_Excel2$n.SNR,
                       mean.c = iNPH_Biochem_TTau_Excel2$mean.SNR,
                       sd.c = iNPH_Biochem_TTau_Excel2$sd.SNR,
                       studlab = iNPH_Biochem_TTau_Excel2$Author,
                       data = iNPH_Biochem_TTau_Excel2,
                       sm = "SMD",
                       method.smd = "Cohen",
                       fixed = FALSE,
                       random = TRUE,
                       method.tau = "REML",
                       hakn = TRUE,
                       title = "T-Tau levels in SR vs N-SR")

#numerical visualisation of meta_ttau (note that SR are the experimental group so reference group, so
minus result means it is less in them)

print(meta_ttau2)

#make the plot

forest.meta(meta_ttau2,
            sortvar = TE,
            predict = TRUE,
            print.tau2 = FALSE,
            leftlabs = c("Author", "g", "SE"),
            lab.e = "S-R",
            lab.c = "S-NR",
            JAMA.pval = FALSE,
            test.overall.random = TRUE,
            label.test.overall.random = "Overall statistical result of model: ")
```

```
##############################################################################################################

# Load NFL dataset from dmetar (or download and open manually)

library(readxl)
iNPH_Biochem_NFL_Excel <- read_excel("~/Desktop/iNPH Biochem - NFL.xlsx")
View(iNPH_Biochem_NFL_Excel)

# Use metcont to pool results.
meta_NFL <- metacont(n.e = iNPH_Biochem_NFL_Excel$n.SR,
                     mean.e = iNPH_Biochem_NFL_Excel$mean.SR,
                     sd.e = iNPH_Biochem_NFL_Excel$sd.SR,
                     n.c = iNPH_Biochem_NFL_Excel$n.SNR,
                     mean.c = iNPH_Biochem_NFL_Excel$mean.SNR,
                     sd.c = iNPH_Biochem_NFL_Excel$sd.SNR,
                     studlab = iNPH_Biochem_NFL_Excel$Author,
                     data = iNPH_Biochem_NFL_Excel,
                     sm = "SMD",
                     method.smd = "Cohen",
                     fixed = FALSE,
                     random = TRUE,
                     method.tau = "REML",
                     hakn = TRUE,
                     title = "NFL levels in SR vs N-SR")

#numerical visualisation of meta_ttau (note that SR are the experimental group so reference group, so
minus result means it is less in them)

print(meta_NFL)

#make the plot

forest.meta(meta_NFL,
            sortvar = TE,
            predict = TRUE,
```

```
            print.tau2 = FALSE,
            leftlabs = c("Author", "g", "SE"),
            lab.e = "S-R",
            lab.c = "S-NR",
            JAMA.pval = FALSE,
            test.overall.random = TRUE,
            label.test.overall.random = "Overall statistical result of model: ")


#########################################################################################################
##############



# Load Sulfatide dataset from dmetar (or download and open manually)

library(readxl)
iNPH_Biochem_Sulfatide_Excel <- read_excel("~/Desktop/iNPH Biochem - Sulfatide.xlsx")
View(iNPH_Biochem_Sulfatide_Excel)

# Use metcont to pool results.
meta_Sulfatide <- metacont(n.e = iNPH_Biochem_Sulfatide_Excel$n.SR,
                           mean.e = iNPH_Biochem_Sulfatide_Excel$mean.SR,
                           sd.e = iNPH_Biochem_Sulfatide_Excel$sd.SR,
                           n.c = iNPH_Biochem_Sulfatide_Excel$n.SNR,
                           mean.c = iNPH_Biochem_Sulfatide_Excel$mean.SNR,
                           sd.c = iNPH_Biochem_Sulfatide_Excel$sd.SNR,
                           studlab = iNPH_Biochem_Sulfatide_Excel$Author,
                           data = iNPH_Biochem_Sulfatide_Excel,
                           sm = "SMD",
                           method.smd = "Cohen",
                           fixed = FALSE,
                           random = TRUE,
                           method.tau = "REML",
                           hakn = TRUE,
                           title = "Sulfatide levels in SR vs N-SR")

#numerical visualisation of meta_ttau (note that SR are the experimental group so reference group, so
minus result means it is less in them)
```

```
print(meta_Sulfatide)

#make the plot

forest.meta(meta_Sulfatide,
            sortvar = TE,
            predict = TRUE,
            print.tau2 = FALSE,
            leftlabs = c("Author", "g", "SE"),
            lab.e = "S-R",
            lab.c = "S-NR",
            JAMA.pval = FALSE,
            test.overall.random = TRUE,
            label.test.overall.random = "Overall statistical result of model: ")



##########################################################################################################
##############



# Load Total set dataset from dmetar (or download and open manually)

library(readxl)
iNPH_Biochem_Total_Excel <- read_excel("~/Desktop/iNPH Biochem - Total.xlsx")
View(iNPH_Biochem_Total_Excel)

# Use metcont to pool results.
meta_Total <- metacont(n.e = iNPH_Biochem_Total_Excel$n.SR,
                       mean.e = iNPH_Biochem_Total_Excel$mean.SR,
                       sd.e = iNPH_Biochem_Total_Excel$sd.SR,
                       n.c = iNPH_Biochem_Total_Excel$n.SNR,
                       mean.c = iNPH_Biochem_Total_Excel$mean.SNR,
                       sd.c = iNPH_Biochem_Total_Excel$sd.SNR,
                       studlab = iNPH_Biochem_Total_Excel$Author,
                       data = iNPH_Biochem_Total_Excel,
                       sm = "SMD",
```

```
                       method.smd = "Cohen",
                       fixed = FALSE,
                       random = TRUE,
                       method.tau = "REML",
                       hakn = TRUE,
                       title = "Total levels in SR vs N-SR")

#numerical visualisation of meta_ttau (note that SR are the experimental group so reference group, so
minus result means it is less in them)

print(meta_Total)

#make the plot

forest.meta(meta_Total,
            sortvar = TE,
            predict = TRUE,
            print.tau2 = FALSE,
            leftlabs = c("Author", "g", "SE"),
            lab.e = "S-R",
            lab.c = "S-NR",
            JAMA.pval = FALSE,
            test.overall.random = TRUE,
            label.test.overall.random = "Overall statistical result of model: ")


#Eggers (Publication bias) calculatiom

eggersplot <-metabias(
  meta_Total,
  method.bias = meta_Total$method.bias,
  plotit = FALSE,
  correct = FALSE,
  k.min = 10
)

print(eggersplot,
      digits = gs("digits"),
      digits.stat = gs("digits.stat"),
```

```
      digits.pval = max(gs("digits.pval"), 2),
      digits.se = gs("digits.se"),
      digits.tau2 = gs("digits.tau2"),
      scientific.pval = gs("scientific.pval"),
      big.mark = gs("big.mark"),
      zero.pval = gs("zero.pval"),
      JAMA.pval = gs("JAMA.pval"),
      text.tau2 = gs("text.tau2"))



#Eggers (Publication bias) plot


eggersplot <-metabias(
  meta_Total,
  method.bias = meta_Total$method.bias,
  plotit = TRUE,
  correct = FALSE,
  k.min = 14
)

funnel.meta(meta_Total,
            xlim = c(-2, 1),
            studlab = FALSE,
            method.bias = "linreg")
```

Supplementary Table 4: The data spreadsheet that provided the basis for meta-analysis and meta-regression for Total-Tau

Author	mean.SR	sd.SR	n.SR	mean.SNR	sd.SNR	n.SNR	age	females	sample	date	srm	neuro	dropout	SMD	SE
Migliorati et al.	161.1	185.6	15	245	137.975	35	75.3	0.32	50	2021	1	0	0	-0.55	0.313776
Tarnaris et al.	550.97	551.65	17	1086	347.19	4	71.45	0.32	21	2011	0	1	0	-1.02	0.576531
Ågren-Wilsson et al.	164	115	33	269	93	22	72	0.37	55	2007	0	1	0	-0.98	0.290816
Hong et al.	141.9	40.8	17	177.2	126.7	14	78.7	0.35	31	2018	1	0	1	-0.39	0.364796
Vanninen et al.	190.4	86.8	91	211.3	75.9	27	75.4576		118	2021	1	0	0	-0.25	0.219388
Tullberg et al.	267	296	28	275	165	7	68	0.636	18	2008	0	0	0	-0.03	0.423469

Supplementary Table 5: The data spreadsheet that provided the basis for meta-analysis and meta-regression for Total-Tau

Author	mean.SR	sd.SR	n.SR	mean.SNR	sd.SNR	n.SNR	age	females	sample	date	srm	neuro	dropout	SMD	SE
Migliorati e	26.6	13.75	15	39.6	14.325	35	75.3	0.32	50	2021	1	0	0	-0.92	0.321429
Ågren-Wils	32	10	33	37	12	22	72	0.37	55	2007	0	1	0	-0.85	0.278061
Hong et al.	29.8	12.9	17	47.6	27.8	14	78.7	0.35	31	2018	1	0	1	-0.46	0.377551
Vanninen e	34.7	14.8	91	38.3	12.2	27	75.4576		118	2021	1	0	0	-0.25	0.219388

Supplementary Table 6: The data spreadsheet that provided the basis for meta-analysis and meta-regression for Amyloid-beta 1-12

Author	mean.SR	sd.SR	n.SR	mean.SNR	sd.SNR	n.SNR	age	females	sample	date	srm	neuro	dropout	SMD	SE
Migliorati e	513.3	254.95	15	793	299.925	35	75.3	0.32	50	2021	1	0	0	-0.97	0.32398
Ågren-Wils	515	107	33	492	98	22	72	0.37	55	2007	0	1	0	0.22	0.27551
Hong et al.	581	173.9	17	594.3	274.3	14	78.7	0.35	31	2018	1	0	1	-0.06	0.362245
Vanninen e	673.9	192.2	91	617.5	172.7	27	75.4576		118	2021	1	0	0	-0.1	0.471939

References

1. Ågren-Wilsson A, Lekman A, Sjöberg W, Rosengren L, Blennow K, Bergenheim AT, Malm J (2007) CSF biomarkers in the evaluation of idiopathic normal pressure hydrocephalus. Acta Neurol Scand 116(5):333-9
2. Hong YJ, Kim MJ, Jeong E, Kim JE, Hwang J, Lee JI, Lee JH, Na DL (2018) Preoperative biomarkers in patients with idiopathic normal pressure hydrocephalus showing a favorable shunt surgery outcome. J Neurol Sci 387:21-26
3. Migliorati K, Panciani PP, Pertichetti M, Borroni B, Archetti S, Rozzini L, Padovani A, Terzi L, Bruscella S, Fontanella MM (2021) P-Tau as prognostic marker in long term follow up for patients with shunted iNPH. Neurol Res 43(1):78-85
4. Page MJ, McKenzie JE, Bossuyt PM, Boutron I, Hoffmann TC, Mulrow CD, Shamseer L, Tetzlaff JM, Akl EA, Brennan SE, Chou R, Glanville J, Grimshaw JM, Hróbjartsson A, Lalu MM, Li T, Loder EW, Mayo-Wilson E, McDonald S, McGuinness LA, Stewart LA, Thomas J, Tricco AC, Welch VA, Whiting P, Moher D (2021) The PRISMA 2020 statement: an updated guideline for reporting systematic reviews. BMJ 372:n71
5. Sterne JA, Hernán MA, Reeves BC, Savović J, Berkman ND, Viswanathan M, Henry D, Altman DG, Ansari MT, Boutron I, Carpenter JR, Chan AW, Churchill R, Deeks JJ, Hróbjartsson A, Kirkham J, Jüni P, Loke YK, Pigott TD, Ramsay CR, Regidor D, Rothstein HR, Sandhu L, Santaguida PL, Schünemann HJ, Shea B, Shrier I, Tugwell P, Turner L, Valentine JC, Waddington H, Waters E, Wells GA, Whiting PF, Higgins JP (2016) ROBINS-I: a tool for assessing risk of bias in non-randomised studies of interventions. BMJ 355:i4919
6. Tarnaris A, Toma AK, Chapman MD, Keir G, Kitchen ND, Watkins LD (2011) Use of cerebrospinal fluid amyloid-β and total tau protein to predict favorable surgical outcomes in patients with idiopathic normal pressure hydrocephalus. J Neurosurg 115(1):145-50
7. Tullberg M, Blennow K, Månsson JE, Fredman P, Tisell M, Wikkelsö C (2008) Cerebrospinal fluid markers before and after shunting in patients with secondary and idiopathic normal pressure hydrocephalus. Cerebrospinal Fluid Res 5:9
8. Vanninen A, Nakajima M, Miyajima M, Rauramaa T, Kokki M, Musialowicz T, Mäkinen PM, Herukka SK, Koivisto AM, Jääskeläinen JE, Hiltunen M, Leinonen V (2021) Elevated CSF LRG and Decreased Alzheimer's Disease Biomarkers in Idiopathic Normal Pressure Hydrocephalus J Clin Med 10(5):1105
